# Abstracts of the 9^th^ International Conference on Cachexia, Sarcopenia, and Muscle Wasting, Berlin, Germany, 10–11 December 2016 (part 2)

**DOI:** 10.1002/jcsm.12182

**Published:** 2017-02-27

**1‐39**

**A review of body composition, bone mineral density, and anthropometry in a cohort of 2521 women presenting for bone mineral density testing in a tertiary centre**

Boyd J. G. Strauss ^1^, Ming Li Yee^2^ and Christopher Gilfillan^2^

^1^ *Department of Medicine, School of Clinical Sciences, Monash University Clayton, Victoria, Australia;* ^2^ *Endocrinology Department Eastern Clinical School, Eastern Health, Melbourne, Victoria, Australia*

**Background**: Sarcopenia is defined as loss of skeletal muscle mass and function. In clinical research, measurement of skeletal muscle mass index (SMI) is based on appendicular skeletal muscle mass (kg)/height ^2^ (m^2^) <2 SD below the mean of a young reference group.^1^ SMI was found to be associated with increased physical disability independent of age, ethnicity, comorbidity, and fat mass.^2^ Alternatively, SMI is reported as weight‐adjusted SMI derived from values obtained from bioelectrical impedance analysis.^3^ A recent study suggests the height‐adjusted SMI has better correlation with reduced muscle strength and gait speed.^4^

**Methods**: We assessed data on 2521 women with a variety of medical conditions who presented to a tertiary hospital for assessment of bone mineral density (BMD). Some of these women also had concurrent body composition and anthropometry performed.

**Aims**: We aim

- To determine the relationship between BMD, body composition, and anthropometry in the whole group and subgroups.
- To determine The relationship between age and decline in skeletal muscle mass and its relationship to anthropometric variables.
- To explore leg length (which appears constant with age) vs. height (which declines with age) in standardizing skeletal muscle measurement for body size.

**Results**: Participants were females aged 40–97 years (mean 59 years). The majority of the patients who were referred presented on a background of breast cancer (*n* = 679, 27.9%), followed by screening or other causes (*n* = 563, 22.3%) and steroid use (*n* = 241, 9.6%).

When analysed on regression variable plots, reduction in sitting height, biceps, thigh skin fold, total body, femoral neck BMD, and T score occurs with age.

**Discussion**: There were no changes observed in leg length with age. This suggests that leg length may be used as an alternative to height in the calculation of skeletal muscle mass index.

1. Baumgartner RN, et al Epidemiology of sarcopenia among the elderly in New Mexico, American Journal of Epidemiology 147:8: 755‐763
2. Cruz –Jentoft AJ et al, Sarcopenia: European consensus on definition and diagnosis Report of the European Working Group on Sarcopenia in Older People Age and Ageing 2010: 39: 412‐423
3. Janssen et al, Low relative skeletal muscle mass (sarcopenia) in older persons is associated with functional impairment and physical disability. J. Am. Geriatr. Soc. 50, 889–896 (2002)
4. Han DS et al, Skeletal muscle mass adjusted by height correlated better with muscular functions than that adjusted by body weight in defining sarcopenia Scientific Reports 20 January 2016 1‐8

**1-40**

**Low muscle mass at initiation of anti‐tumour necrosis factor therapy for inflammatory bowel disease is associated with early treatment failure**

Darcy Quinn Holt^1,2^, Poornima Varma^1^, Boyd Josef Gimnicher Strauss ^2^, Anton S. Rajadurai^2^ and Gregory Thomas Moore^1,2^

^1^ *Department of Gastroenterology and Hepatology, Mon ash Health, Clayton, Victoria, Australia;* ^2^ *School of Clinical Sciences at Monash Health, Monash University, Clayton, Victoria, Australia*

**Goals**: We sought to determine whether low muscle mass at commencement of anti‐tumour necrosis factor (TNF) therapy was associated with earlier treatment failure.

**Background**: Delayed treatment failure occurs in significant proportion of inflammatory bowel disease (IBD) patients treated with TNF‐alpha antagonists. Identification of predictors of loss of response may help optimize therapy.

**Study**: A retrospective cohort study was performed of 67 patients who had undergone cross‐sectional abdominal imaging at a single centre coincident with commencement of anti‐TNF drugs. Analysis of the images at the third lumbar vertebra was performed using standard techniques to determine cross‐sectional areas of skeletal muscle (SM), visceral adipose tissue, subcutaneous adipose tissue, and intermuscular adipose tissue. Treatment failure was defined as follows: a post‐induction hospital admission or surgery for IBD, escalation of TNF dose or immunosuppressants for clinical loss of response, emergence of a new fistula, or rising Crohn's Disease Activity Index >150.

**Results**: Two‐thirds of patients had sarcopenia. Patients with less than the gender‐specific median SM area had a median time to failure of 520 (SD 135) days compared with 1100 (SD 151) days for those with greater than median SM area (*P* = 0.036). No difference was found in disease duration, inflammatory markers, or Crohn's Disease Activity Index between quartiles of SM area. No relation between outcomes and measures of adipose tissue, weight, or body mass index was observed.

**Conclusions**: Identifying low muscle mass at anti‐TNF induction as a risk factor for treatment failure may contribute to a more tailored approach to IBD therapy.

**1-41**

**Cushing's syndrome: a model for sarcopenic obesity**

Michael Drey*, Christina M. Berr, Martin Reincke, Julia Fazel, Jochen Seissler, Jochen Schopohl, Martin Bidlingmaier, Stefanie Zopp, Nicole Reisch, Felix Beuschlein, Andrea Osswald and Ralf Schmidmaier

*Medizinische Klinik und Poliklinik IV, Klinikum der Universität München (LMU), Munich, Germany*

**Background and aims**: Obesity and its metabolic impairments are discussed as major risk factors for sarcopenia leading to sarcopenic obesity. Cushing's syndrome (CS) is known to be associated with obesity and muscle atrophy. The German Cushing Registry prospectively studies phenotypic and biochemical characteristics of CS. We compared CS with matched obese controls (OC) regarding body composition, physical performance, and biochemical markers to test the hypothesis that CS could be a model for sarcopenic obesity.

**Methods**: Analysed were 47 patients with active CS and 108 controls. By propensity score matching, 47 controls were selected by body mass index and gender as OC. Fat mass and muscle mass were measured by bioelectrical impedance analysis. Muscle function was assessed by chair rising test and hand grip strength. Multiple regression analysis was used to investigate differences in body composition, physical performance, and biochemical markers adjusted for gender and age between the groups.

**Results**: Muscle mass did not differ between CS and OC (25.3 kg vs. 25.6 kg, *P* = 0.793). However, CS patients showed significantly greater chair rising time (9.5 s vs. 7.3 s, *P* = 0.008) and significantly lower hand grip strength (32.1 kg vs. 36.8 kg, *P* = 0.003). Furthermore, waist‐to‐hip ratio (1.0 vs. 0.9, *P* < 0.001) and HbA1c (6.1% vs. 5.4%, *P* < 0.001) adjusted for age and gender were higher in CS. CS patients with impaired fasting glucose have shown the highest limitations in hand grip strength (*P* = 0.025) and chair rising time (*P* < 0.001).

**Conclusions**: Similar to published data in geriatrics, CS patients show impaired muscle function that cannot be explained by low muscle mass. Impaired muscle quality due to fat infiltration may be the reason. Research in sarcopenic obesity in elderly is hampered by confounding comorbidities and polymedication. As CS patients are frequently free of comorbidities and as CS is potentially curable, we suggest CS as a clinical model for further research in sarcopenic obesity.

**1-42**

**Serum creatinine/cystatin c ratio predicts strongly both sarcopenia and poor prognosis in the elderly**

Takayuki Tsuneda, Hidehiko Nagasawa, Atsuhiro Shimakura and Masanobu Takata

*Internal Medicine, Toyama Teishin Hospital, Toyama, Japan*

**Background**: Sarcopenia develops poor prognosis in the elderly. Serum creatinine (SCr) is a major biomarker to reflect not only renal functio, but also total mass of muscle. Recently, cystatin C (CysC) is building up a renal marker without any influences of muscle mass and superior to SCr to estimate renal insufficiency. Previous reports show that low value of SCr did not reflect reserved renal function, especially in lean elderly woman with malnutrition. Therefore, we evaluated retrospectively the impact of SCr/CysC ratio for sarcopenia, the activities of daily living (ADLs), and the prognosis.

**Method**: We enrolled 324 patients in our hospital (79 ± 13 years old; men, 46%) and collected SCr, CysC, and albumin. For estimation of sarcopenia, we measured the area of bilateral psoas muscle normalized by height [total psoas index (TPI), mm^2^/m^2^] and the Hounsfield unit average calculation (HUAC, HU) as a marker of muscle density and fatty infiltration, using computed tomography (*n* = 195). ADL was classified by modified Rankin scale (mRS). We also analysed mortality outcome based on SCr/CysC ratio.

**Results**: Serum creatinine/CysC ratio decreased with aging (*P* < 0.0001). TPI in female was smaller than that in male (*P* < 0.0001), without gender difference in HUAC. TPI associated with body weight (*R* ^2^ = 0.41), and HUAC did albumin (*R* ^2^ = 0.23) and SCr/CysC ratio (*R* ^2^ = 0.23). The lower SCr/CysC ratio was related to the greater score of mRS (*P* < 0.0001) with decreased HUAC (*P* < 0.0001). SCr/CysC ratio was superior in predicting the lower ADL (mRS ≥ 3) with 82.4% of sensitivity and 70.4% of specificity (cut‐off value, 0.705) to TPI or HUAC, assessed by receiver operating characteristic analysis (area under the curve: 0.83, 0.60, and 0.73, respectively). According to Kaplan–Meier analysis, the low SCr/CysC ratio (<0.70) associated with unfavourable prognosis because of all causes (*P* < 0.0001).

**Conclusion**: Our study indicated that the SCr/CysC ratio might predict poor prognosis as well as the degree of sarcopenia in the elderly.

**1-43**

**Sarcopenia, vitamin D, and functional status in mild Alzheimer's disease and community‐dwelling older adults**

Odete Vicente de Sousa ^1,2,3^ and Teresa Freitas do Amaral^1,4^

^1^ *Faculdade de Ciências da Nutrição e Alimentação, Universidade do Porto, Porto, Portugal;* ^2^ *UNIFAI – Instituo de Ciências Biomédicas Abel Salazar, Universidade do Porto, Porto, Portugal;* ^3^ *Hospital de Magalhães Lemos E.P.E., Porto, Portugal;* ^4^ *UISPA – LAETA/INEGI, Faculdade de Engenharia, Universidade do Porto, Porto, Portugal*

**Background**: The knowledge on the association of sarcopenia, vitamin D, and functional status with Alzheimer's disease (AD) is limited. The present study aims to evaluate the association of sarcopenia, vitamin D, and functional factors with mild AD.

**Methods**: A case–control study was conducted among 79 mild AD community‐dwelling older adults (32 men; age: 78.2 ± 6.6 years) and 32 non‐AD community‐dwelling older adults (seven men; age: 72.3 ± 7.4 years). Nutrition status was assessed using MNA® score, serum 25‐hydroxyvitamin D3 [25(OH)D3] and bioimpedance analysis. Sarcopenia was defined according to EWGSOP 2010 criteria. Functional status using gait speed, handgrip strength, and Barthel index was determined. Multivariate backward logistic regression analyses were carried out.

**Results**: Thirty‐three AD patients (41.7%) and three (9.3%) controls showed sarcopenia. Among the sarcopenic AD patients, 54.5% were classified as undernourished and the remaining at undernutrition risk. All the sarcopenic controls were non‐undernourished. Sixty‐five AD patients (82.3%) and 20 (62.5%) controls had serum 25(OH)D3 deficiency (20.1–30.0 ng/mL). Sarcopenia [OR = 7.75, 95% confidence interval (CI) 1.59–37.74, *P =* 0.011], gait speed (OR 16.94, CI 95% 3.93–72.90, *P* < 0.001), and Barthel index (OR 7.93, CI 95% 2.41–26.06, *P* = 0.001) were associated with AD.

**Conclusion**: A high proportion of AD patients (95%) and controls (90.6%) had 25(OH)D3 insufficiency (<30 ng/mL). Sarcopenia, low gait speed, and dependence were strongly associated with AD.

**Conflict of interest**: The authors have reported no conflicts of interest. This article has no sponsorship.

**1-44**

**Electronically administered patient reported outcomes (ePROs) in sarcopenic older patients: the SARA clinical data platform novel approach to clinical trials**

Susanna Del Signore ^1,2^, Gianluca Zia^1^, Stefania Del Signore^1^ and Waly Dioh^2^

^1^ *Bluecompanion ltd, London, UK;* ^2^ *Biophytis, Romainville, France*

Sarcopenia was recently recognized as an independent condition by the International Classification of Disease, Tenth Revision, Clinical Modification under the code M62.84.

Although no candidate drug has received yet marketing authorization for (ageing related) sarcopenia, US and EU regulatory experts do strengthen the importance of testing patient‐reported outcomes aside from objective measurements of mobility function and body composition, to demonstrate clinical efficacy in sarcopenia.

**Rationale**: Standard designed clinical trials may be inadequate for collecting good quality long‐term clinical data in older adults. Concomitant chronic diseases and poly‐therapy‐related exclusion criteria often prevent enrolling a representative sample of the target population. Rigid visit and investigation scheduling negatively affects compliance and retention rate while increasing the risk of biased results.

Administering PROs to older patients via remotely connected devices as an approach for good quality clinical data is now being tested in SARA‐OBS, an EU/US study assessing the 6 month rate of change in physical function and body composition of 300 community dwelling older patients with low SPPB, at risk of mobility disability and physical dependence.

SARA clinical data platform is based on a semi‐permanent clinical trial infrastructure, centred on geographic areas and their clinical investigation centres. A digital platform integrates different source data: clinical data, DXA, physical activity recording, biomarkers (via a Biobank), and PROs. At study end, data could be retrieved for secondary research in sarcopenia, for example, answering additional, not prespecified questions from regulators.

The clinical trial platform is enabled by novel information and communication technologies, allowing friendly secure communication with patients and continuous data collection from home, minimizing travels to the investigational site. This approach succeeds to empower patient's role in clinical research by providing simplified communication with the study staff. A dedicated website as entry portal and an adaptive data warehouse complete the SARA clinical data platform infrastructure.

**1-45**

**Psoas muscle measurements are inferior to total skeletal muscle measurements in the assessment of sarcopenia in ovarian cancer**

Iris J. Rutten^1^, Jorne Ubachs ^2^, Roy F. P. M. Kruitwagen^2^, Regina G. H. Beets‐Tan^1^, Steven W. M. Olde Damink^3^ and Toon van Gorp^2^

^1^ *Department of Radiology, Maastricht University Medical Centre, Maastricht, The Netherlands;* ^2^ *Department of Obstetrics and Gynaecology, Maastricht University Medical Centre, Maastricht, The Netherlands;* ^3^ *Department of General Surgery, Maastricht University Medical Centre, Maastricht, The Netherlands*

**Background**: Computed tomography measurements of total skeletal muscle area can detect changes and predict overall survival (OS) in patients with advanced ovarian cancer. This study investigates whether assessment of psoas muscle area reflects total muscle area and data collected can be used to assess sarcopenia in ovarian cancer patients.

**Methods**: Ovarian cancer patients (*n* = 150) treated with induction chemotherapy and interval debulking were enrolled retrospectively in this longitudinal study. Muscle was measured cross‐sectionally with computed tomography in three ways: (i) software quantification of total skeletal muscle area (SMA); (2) software quantification of psoas muscle area (PA); and (3) manual measurement of length and width of the psoas muscle to derive the psoas surface area (PLW). Pearson correlation between the different methods was studied. Patients were divided into two groups based on the extent of change in muscle area, and agreement was measured with kappa coefficients. Cox regression was used to test predictors for OS.

**Results**: Correlation between SMA and both psoas muscle area measurements was poor (*r* = 0.52 and 0.39 for PA and PLW, respectively). After categorizing patients into muscle loss or gain, kappa agreement was also poor for all comparisons (all κ < 0.40). In regression analysis, SMA loss was predictive of poor OS [hazard ratio 1.698 (95%CI 1.038–2.778), *P* = 0.035]. No relationship with OS was seen for PA or PLW loss.

**Conclusions**: Change in psoas muscle area is not representative of the total muscle area change and should not be used to substitute total skeletal muscle to predict survival in patients with ovarian cancer.

**1-46**

**Loss of lean mass after stroke: results from the prospective observational study on body composition in acute stroke (BoSSS)**

Charlotte Pietrock ^1^, Nadja Scherbakov^1,2^, Jochen B. Fiebach^1^, Nicole Ebner^3^, Anja Sandek^3^, Miroslava Valentova^3^, Stephan von Haehling^3^, Stefan D. Anker^3^ and Wolfram Doehner^1,2,4^

^1^ *Center for Stroke Research Berlin (CSB), Charité University Medical School, Berlin, Germany;* ^2^ *German Center for Cardiovascular Diseases (DZHK), Partner Site, Berlin;* ^3^ *Innovative Clinical Trials, Department of Cardiology and Pneumology University Medical Centre Göttingen, Germany;* ^4^ *Department of Cardiology, Charité University Medical School, Berlin, Germany*

**Introduction**: The structural and functional deterioration of skeletal muscle in paretic stroke patients has a strong impact on clinical outcome after stroke. Using dual‐energy X‐ray absorptiometry (DEXA) to analyse changes in body composition, this study aimed to assess long‐term loss and remodelling processes of skeletal muscles of stroke patients.

**Methods**: A total of 131 patients with acute ischemic stroke who were admitted to a university centre and stroke unit (age: 67 ± 13 years; 80 men, 51 women; BMI: 27 ± 4 kg/m^2^, mean NIHSS: 4 ± 3) were studied in a prospective longitudinal observational study. DEXA was used to assess fat and lean tissue, and appendicular lean mass of the affected (paralytic) vs. non‐affected body side was studied. DEXA studies were performed immediately at the time of acute stroke (4 ± 2 days) and at 1 (12.8 ± 0.9 months, *n* = 67) and 2 year (24.9 ± 1.3 months, *n* = 44) follow‐up (FU). Patients (34%, *n* = 44) with acute stroke received thrombolytic therapy at admission to the hospital. Volume of early ischemic injury was analysed using the quantitative topographic Alberta stroke programme early CT score (ASPECTS), which revealed an average of 8.6 ± 1.4 points.

**Results**: The baseline examination showed a trend towards reduction of lean mass of the paretic arms and/or legs compared with the non‐paretic arms and/or legs (*P* = 0.0551). Significantly less lean mass was found in the paretic arms and/or legs compared with the non‐paretic arms and/or legs at 1 and 2 year FU (both *P* < 0.001). There was no significant change in lean mass in the non‐paretic arms and/or legs at 1 and 2 year FU (*P* = 0.4198 and *P* = 0.3484, respectively). Additionally, at 1 and 2 year FU, reduction of BMI was observed in patients without thrombolytic therapy (both *P* < 0.001). In contrast, no reduced BMI and no loss of lean mass were observed during 2 year FU in patients who received thrombolytic therapy (all *P* > 0.16). Patients without thrombolytic therapy showed significantly less lean mass in the paretic arms and/or legs compared with that in the non‐paretic arms and/or legs (*P* = 0.009). At 1 year FU, patients with an initial ASPECTS ≤8 points, indicating a higher volume of brain injury, showed significantly less lean mass in the paretic arms and/or legs compared with that in the non‐paretic arms and/or legs in contrast with patients with a score of ≥9 points (*P* < 0.05).

**Conclusion**: Muscle wasting was observed in patients after acute stroke in the paretic limb, but not in the non‐paretic limb, and was continuously observed after 2 years of FU. Tissue wasting was particularly present in patients without thrombolytic therapy and in this with higher volume of brain injury.

**1-47**

**The influence of operative stress, systemic inflammation, and sepsis on computed tomography body composition variables**

Michael Ramage ^1^, Graeme W. Couper^2^, James A. Ross^3^, Christopher D.A. Deans^1^, Richard J. E. Skipworth^1^ and Kenneth C. H. Fearon*^1^

^1^ *Clinical Surgery, Royal Infirmary of Edinburgh, Edinburgh, UK;* ^2^ *NHS Lothian, Royal Infirmary of Edinburgh, Edinburgh, UK;* ^3^ *Tissue Injury and Repair Group, Chancellor's Building, University of Edinburgh, Edinburgh, UK*

**Introduction**: Computed tomography (CT) planimetry at the L3 level can identify sarcopenia and prognosticate morbidity and mortality in cancer patients. The deleterious impact of neoadjuvant chemotherapy on CT‐derived lean body mass has been assessed previously. However, the impact of systemic inflammation (SI) on CT‐derived body composition variables has not been investigated.

**Methods**: Pre‐operative staging and post‐operative portal venous phase CT scans of oesophageal cancer patients who underwent Ivor Lewis esophagectomy and then subsequently developed sepsis secondary to anastomotic leak (*n* = 14; M : F 12 : 2; median age 67 years) were analysed. Body composition variables were collected using validated semi‐automated analysis software (Matlab).

**Results**: There were no significant differences in L3 skeletal muscle area [median 149.7 cm^2^ (range 82.5–203.2) vs. 156.1 cm^2^ (112.6–205.5); *P* = 0.11] or subcutaneous adipose tissue area [210.4 cm^2^ (93.1–696.9) vs. 219.2 cm^2^ (97.8–346.2); *P* = 0.55] between pre‐ and post‐operative scans. There was a significant reduction in visceral adipose tissue area [179.0 cm^2^ (51.7–380.5) vs. 154.1 cm^2^ (40.7–364.9); *P* = 0.041], but this is explained by the removal of the surgical specimen. However, there was a significant reduction in skeletal muscle density (in Hounsfield units) between pre‐and post‐operative scans [36.8 (20.0–49.7) vs. 27.5 (15.5–46.1); *P* = 0.026], whereas there were significant increases in both visceral adipose tissue density (−97.7 [−104.8 to −74.6] vs. −88.8 [−98.6 to −71.30]; *P* = 0.002) and subcutaneous adipose tissue density (−102.77 [−111.71 to −87.86] vs. −86.44 [−97.00 to −71.14]; *P* = 0.001).

**Conclusion**: The lack of change in skeletal muscle area between pre‐ and post‐operative scans would support the reliability of L3 CT planimetry to assess sarcopenia across repeated assessments. In the early phase, operative stress and SI (sepsis) are associated with alterations in tissue density, likely explained by an increase in tissue oedema and/or altered blood flow affecting contrast uptake. Care must be taken when interpreting skeletal muscle density/myosteatosis in the presence of SI, as CT‐derived body composition will be affected by the patient's condition.

**1-48**

**Albuminuria and hemodynamics in patients with heart failure referred for transplantation**

Piotr Rozentryt ^1^, Jacek Niedziela^1^, Jolanta U. Nowak^1^, Bartosz Hudzik^1^, Marek Gierlotka^1^, Andrzej Lekston^1^, Michał Hawranek^1^, Ewa Jankowska^2^, Stephan von Haehling^3^, Wolfram Doehner^3^, Stefan D. Anker^3^ and Mariusz Gąsior^1,4^

^1^ *III Department of Cardiology, Silesian Centre for Heart Disease, Silesian Medical University, Zabrze, Poland;* ^2^ *Department of Heart Diseases, Wroclaw Medical University, Wroclaw, Poland;* ^3^ *Department of Innovative Clinical Trials, University Medical Center Göttingen (UMG), Göttingen, Germany;* ^4^ *Applied Cachexia Research, Department of Cardiology, Charite Medical School—Campus Virchow‐Klinikum, Berlin, Germany*

**Background**: Both albuminuria and reduced kidney function are important prognosticators in heart failure (HF). Reduction of filtration in HF was shown to be linked with elevated venous pressure and low cardiac output. Much less is known on the reasons of for albuminuria in HF. The relations between albumin loss into urine and invasively assessed hemodynamic characteristics have never been studied in HF.

**Aim**: We intended to analyse the relation between degree of albuminuria and hemodynamic characteristics measured invasively using Swan–Ganz catheter.

**Material and methods**: In the 244 patients with HF with reduced ejection fraction referred for qualification to heart transplantation (age: 51 ± 10 years, 12% female: LVEF: 24 ± 11%, NYHA class: 3.0 ± 0.7), we measured mean right atrial (RA), pulmonary artery (PA) and capillary wedge (PCWP) pressures as well as cardiac output (CO) using termodilution technique. Albumin excretion was measured taking advantage of standard method from morning urine sample and expressed in milligrammes per 1 g of excreted creatinine (UA). Clinical, laboratory, and hemodynamic characteristics of patients with different UA values were compared. We used logistic regression to estimate the association of various variables on the risk of UA above median value as compared with UA bellow median.

**Results**: The median UA was 1.64 mg/g of creatinine. The group with higher UA was not different with respect to age, BMI, NYHA class, LVEF, hsCRP, NTproBNP, MDRD, and per cent‐recommended dosages of beta‐blockers, ACEI/ARB, and aldosterone antagonist. The group with UA above median had higher RA (8 ± 3 vs. 3 ± 1 mmHg, *P* < 0.0001), PA (30 ± 11 vs. 23 ± 9 mmHg, *P* = 0.01), and PCWP (20 ± 10 vs. 14 ± 7 mmHg, *P* < 0.0001) higher dose of furosemide equivalent (121 ± 75 vs. 101 ± 57 mg). There was also more women in this group. The CO was not different (5.1 ± 4.2 vs. 4.6 ± 2.2 L/min, *P* = 0.69).

In univariate analysis, higher UA was associated with sex, OR = 0.60, 95%CI: 0.36–0.99, *P* = 0.04 (male vs. female); weight loss during HF, OR = 2.45, 95%CI: 1.07–5.57, *P* = 0.03 (per 1% increase); NTproBNP, OR = 1.22, 95% CI: 1.08–1.37, *P* = 0.002 (per 1000 pg/mL increase); and dose of loop diuretics, OR = 1.20, 95% CI: 1.00–1.45, *P* = 0.04 (per 40 mg of furosemide equivalent increment).

In multivariable analysis comprising parameters with *P* < 0.2 on univariate analysis, no parameter was predictive of higher UA.

**Conclusion**: Higher albuminuria in patients with HF parallel elevated filling pressures, but we failed to confirm the independent association of these variables with UA.

**1-49**

**Fractional urinary excretion of potassium, treatment pattern, and mortality in heart failure**

Piotr Rozentryt ^1^, Jacek Niedziela^1^, Jolanta U. Nowak^1^, Bartosz Hudzik^1^, Przemysław Leszek^2^, Tomasz Rywik^2^, Ewa Jankowska^2^, Wolfram Doehner^3^, Stephan von Haehling^4^, Stefan D. Anker^4^ and Mariusz Gąsior^1^

^1^ *III Department of Cardiology, Silesian Centre for Heart Disease, Silesian Medical University, Zabrze, Poland;* ^2^ *Department of Heart Failure and Transplantology, Institute of Cardiology, Warsaw, Poland;* ^3^ *Applied Cachexia Research, Department of Cardiology, Charite Medical School—Campus Virchow‐Klinikum, Berlin, Germany;* ^4^ *Department of Innovative Clinical Trials, University Medical Center Göttingen (UMG), Göttingen, Germany*

**Introduction**: Fractional urinary excretion of potassium (FUEK) tells us what percentage of potassium filtered within glomerulus is finally lost into urine. It can be taken as a surrogate of renin–angiotensin–aldosterone (RAA) activity on the kidney. In heart failure (HF), RAA system is up‐regulated and degree of activity is associated with poor outcome. In clinical practice, only part of patients receive RAA‐inhibiting therapy at dosages recommended by guidelines. The relation between FUEK, mortality, and treatment quality in this population has never been assessed.

**Aim**: We wanted to determine the association of FUEK and mortality in HF patients treated with various dosages of recommended drugs.

**Material and methods**: In the 380 HF patients (age: 52 ± 11 years, female: 14%, NYHA: 2.7 ± 0.7, LVEF: 24 ± 7%), we measured FUEK from spot morning urine sample. The patients were all treated with angiotensin‐converting enzyme inhibitors (ACEI), beta‐blockers (BB), aldosterone antagonists (AA), and loop diuretics LD at dosages: ACEI: 59 ± 48%, BB: 56 ± 30%, AA: 112 ± 58%, and LD: 101 ± 83 mg of furosemide equivalent. During the follow‐up of 3 years, 106 patients (27.9%) have died. We used Kaplan–Maier method to assess the cumulative probability of death for each tertile of FUEK. Next, we constructed Cox proportional hazard models to estimate the unadjusted risk of death for tertiles of FE and the risk after adjustment for treatment pattern.

**Results**: *Figure* 1 shows Kaplan–Maier curves for mortality at tertiles of FUEK. The hazard ratio of mortality ± 95% confidence intervals for middle and upper tertile of FUEK relative to the bottom in unadjusted and adjusted models are shown in Table 1.

**Table:** 

	Tertiles of FUEK (HR ± 95% CI)		
	Bottom	Middle	Upper
Unadjusted model	1.0	1.59 (0.94–2.68), *P* = 0.08	2.17 (1.33–3.54), *P* = 0.002
Adjusted for MDRD	1.0	1.51 (0.89–2.55), *P* = 0.12	1.74 (1.03–2.94), *P* = 0.03
Adjusted for percent recommended dose of ACEI, BB, AA, the dose of LD, MDRD, and urinary sodium	1.0	1.25 (0.67–2.35), *P* = 0.48	1.79 (0.99–3.24), *P* = 0.052

**Conclusion**: In HF patients, high FUEK may be associated of elevated risk independent of treatment pattern. Whether high FUEK reflects undertreatment remains to be established.

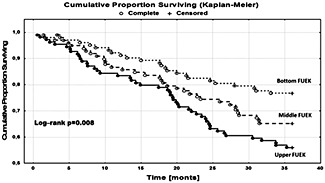

Figure 4 The Kaplan–Maier curves for 3 year mortality in tertiles of fractional urinary excretion of potassium.

**1-50**

**Bioelectrical impedance analysis as an objective nutritional assessment's method in patients undergoing palliative care: preliminary study**

Teresa Małecka‐Massalska ^1^, Monika Prendecka^1^, Radoslaw Mlak^1^, Przemysław Matras^2^, Mariusz Teter^1^, Beata Kolano‐Janeczek^3^ and Waldemar Wójcik^4^

^1^ *Department of Human Physiology, Medical University of Lublin, Lublin, Poland;* ^2^ *Department of General and Transplant Surgery and Nutritional Treatment, Medical University of Lublin, Lublin, Poland;* ^3^ *Hospice of the Good Samaritan in Lublin, Lublin, Poland;* ^4^ *Institute of Electronics and Information Technology, Technical University of Lublin, Lublin, Poland*

**Background and Objective**: Nutritional deficits have a significant impact on mortality, morbidity, and quality of life in cancer patients. Bioelectrical impedance (BIA) has been established as an easy‐to‐use, non‐invasive, reproducible and thus valuable tool in the evaluation of body composition and nutritional status. BIA evaluates body components such as resistance (R) and reactance (Xc) by recording a voltage drop in applied current. The phase angle (PA) calculated as index of capacitance (Xc/R) has been found to be a prognostic indicator in several neoplasms: lung, pancreatic, colon, and breast cancers. This study was conducted to investigate the role of Xc, R, and PA as an malnutrition markers of cancer patients.

**Methods**: We evaluated 12 palliative women (cancer patients hospitalized in Hospice of the Good Samaritan in Lublin, Poland) and 15 healthy volunteers matched by body mass index, age, and sex as a control group—between November 2014 and January 2015. Palliative patients and control group underwent a baseline nutritional assessment: subjective global assessment and BIA. BIA was conducted at 50 kHz.

**Results**: Reactance was found to be significantly lower in palliative patients than in the control group [medians (and 95%CIs) respectively: 2.12 (1.71–2.97) vs. 5.55 (5.18–6.07), *P* < 0.0001]. Similarly, PA was found to be significantly lower in palliative patients than in the control group [medians (and 95%CIs) respectively: 18.55 (9.40–30.69) vs. 49.68 (45.24–53.38), *P* < 0.0001]. No significant differences were found during R analysis: *P* = 0.4945.

**Conclusion**: Palliative patients have altered tissue electrical properties expressed by BIA parameters. BIA could be an alternative method to Subjective Global Assessment measurement and may provide more objectively assess nutritional status of patients with different cancers treated in palliative care units. However, further observations in larger sample sizes are needed to validate use of parameters of BIA as a nutritional marker or prognostic factor in clinical practice.

**1-52**

**Estimation of different body composition parameters in obese patients using bioelectrical impedance**

Nadja Vasiljevic, Dragana Davidovic, Nikolina Banjanin, Branko Jakovljevic, Milos Maksimovic and Jagoda Jorga

*Institute of Hygiene and Medical Ecology, Faculty of Medicine, University of Belgrade, Belgrade, 11000, Serbia*

**Background and Aims**: The assessment of nutritional status is an initial step in the treatment of obese persons. The aim of this study was to evaluate the anthropometric characteristics and body composition variables in relation to sex and age, in obese patients.

**Methods**: The study involved 498 subjects of both sexes who applied to the Department of Nutrition for MNT for obesity. The anthropometric evaluations, as well as the measuremen by using Tanita BC 418 MA bioelectric impedance, were carried out for all patients. On that basis, FM and FFM parameters were obtained, and then the value of FM index (FMI) and FFM index (FFMI) was calculated.

**Results**: The test sample consisted of 76% of females and 24% males. Among them, the highest prevalence was of first degree obesity 37.1%. Body composition analysis has shown that the FFM values differ significantly with respect to these three categories of nutritional status, in both sexes and regarding the age (*P* < 0.001). FFMI values were also significantly different within these three groups (*P* < 0.001) in both sexes. When the differences were analysed in relation to age, it was indicated, in male participants, that with the age increase, FFMI values declined significantly (*P* < 0.02), and they were the lowest in the group of patients older than 60 years. In the oldest group of tested women, the values of FMI were significantly higher (*P* < 0.007).

**Conclusions**: The assessment of nutritional status in people who are on MNT for obesity is necessary, but the estimation of body composition is essential as well, and that fact becomes clear through monitoring FFM and FFMI, as well as FM and FMI. It is particularly important to assess these in the elderly and in order to prevent and monitor sarcopenic obesity.

**1-54**

**Correlation between handgrip strength and muscle mass with biochemical and body composition parameters**

Cristina Garagarza^1^, Ana Laura Flores ^2^ and Ana Valente^1^

^1^ *Nephrocare, Lisbon, Portugal;* ^2^ *Faculty of Medicine, Universidad de Colima, Colima, México*

**Background and Aim**: Hemodialysis (HD) patients are vulnerable to multiple metabolic and nutritional derangements leading to changes in body composition. Several methods to assess muscle reserves have been used; one of this is the handgrip strength (HGS), a simple and reliable method that evaluates muscle strength, and it has been used as a nutritional marker. The aim of this study was to evaluate the correlation of HGS with biochemical parameters and body composition in HD patients.

**Methods**: Single‐centre, cross‐sectional study, where 155 patients in HD were included. Body composition was assessed through bioimpedance spectroscopic. HGS was measured with a hydraulic hand dynamometer in the opposite hand to the vascular access. Protein intake was assessed through normalized protein catabolic rate (nPCR). Albumin and total protein were also evaluated. Data were analysed by sex. *P* value <0.05 was considered statistically significant. IBM SPSS version 20 (IBM, Chicago, IL, USA) was used to perform statistical analysis.

**Results**: Men constitute 60.6% in the study population, and the mean age was 64.4 ± 14.7 years. We found a positive correlation of HGS with lean tissue mass, lean tissue index, and body cell mass and a negative correlation between HGS, age, and OH/ECW in both genders. Albumin presented a positive correlation, and magnesium showed a negative correlation with HGS but only in men. nPCR, total protein, and HD vintage were not correlated with HGS in any of the two groups.

**Conclusions**: Muscle strength is positively correlated with muscle mass; therefore, the muscle strength can be a good marker to determinate changes in muscle mass. Gender influences strength as it is usually higher in men, even in patients in HD, and the HGS tends to decrease with aging. In summary, muscle strength is not only about muscle size; there are other entities that may be associated, as age, sex, and biochemical parameters.

**1-55**

**The desmosomal component, plakoglobin, forms novel complexes in skeletal muscle, whose dissociation promotes atrophy**

Yara Eid Mutlak, Alexandra Volodin, Anna Parnis and Shenhav Cohen

*Technion Institute of Technology, Haifa, Israel*

Skeletal muscle atrophy occurs during fasting and inactivity, naturally with aging, and in many human diseases including diabetes and cancer. During atrophy, there is a major loss of muscle mass and strength, primarily because of the accelerated destruction of the muscle's contractile machinery, the myofibrils, by the proteasome. We have previously shown that plakoglobin is a new constituent of skeletal muscle, which binds to the insulin receptor and the p85 regulatory subunit of PI3K to enhance the insulin signaling, PI3K/Akt cascade, and glucose uptake. Using tandem native purification approach, biochemical fractionation procedures, and mass spectrometry, we now surprisingly demonstrate that plakoglobin forms distinct complexes in skeletal muscle, which contain components of the dystroglycan complex, the insulin receptor and p85/PI3K, and the intermediate filament protein, desmin. Our data indicate that dystroglycan complex integrity is coupled to signal transmission via the insulin receptor and that the desmosomal component plakoglobin is critical for the stability of both structures. Interestingly, in muscles from diabetic mice, where signaling through the IGF‐1 and insulin receptors is impaired, plakoglobin‐containing complexes are compromised. However, overexpression of plakoglobin alone enhanced the stability of these structures. Thus, dissociation of plakoglobin‐containing complexes may be an early event leading to reduced PI3K/Akt signaling, accelerated proteolysis, and atrophy.

**1-56**

**ACE‐2494, a systemically acting transforming growth factor‐β superfamily ligand trap, prevents and restores muscle loss and weakness in disuse atrophy mice**

Jia Li, Maureen Frederics, Rajasekhar N. V. S. Suragani, Scott R. Pearsall and Ravindra Kumar

*Acceleron Pharma, Cambridge, MA, USA*

**Background and Aims**: Skeletal muscle atrophy is a debilitating and a common disorder effecting ~30 million people in the USA.^1^ There is a need for treatments that could accelerate the rate of rehabilitation and reduce overall healthcare costs. We have developed ACE‐2494, a GDF ligand trap that was previously demonstrated to increase muscle mass in wild‐type mice.^2^ This study sought to determine its preventative and therapeutic effects in a model of disuse atrophy mice. Here, an immobilization mouse model was utilized to specifically induce tibialis anterior muscle atrophy and tibiae bone loss.

**Methods**: Thirty‐two 12‐week‐old C57BL/6J male mice were immobilized for 14 days via stapling the unilateral hindlimb. Mice were randomized to receive either vehicle or ACE‐2494 (10 mg/kg) twice per week by subcutaneous delivery. Treatments were carried out either during the 14 day immobilization or during 14 day remobilization. Muscle strength was assessed by performing isometric contraction. Alteration of myocyte morphology was determined by minimal Feret's diameter. Area bone mineral density (aBMD) was measured by dual‐energy X‐ray absorptiometry. All data were normalized to vehicle‐treated unstapled hindlimb to evaluate muscle alterations during immobilization and remobilization.

**Results**: Fourteen‐day immobilization caused a significant reduction in muscle weight, peak tetanic force, myocyte size, and aBMD (−10%, −7%, −17%, and −7%, respectively). Fourteen‐day remobilization was not able to restore the decrease. However, with ACE‐2494 intervention, muscle rehabilitation was greatly accelerated with ~14% muscle hypertrophy accompanied with ~14% higher force generation. Myocyte area was also fully recovered. During the 14 day immobilization, ACE‐2494 treatment resulted in enlarged muscle fibre size by ~28% and restored the loss in aBMD to control levels.

**Conclusions**: ACE‐2494 restored and prevented muscle loss, muscle weakness, and bone loss in disuse atrophy mice. This study provides proof of concept for potential treatments against human disorders involving muscle atrophy.

Reference:1.Dyle MC, et al. (2014) Systems‐based discovery of tomatidine as a natural small molecule inhibitor of skeletal muscle atrophy. J Biol Chem 289(21):14913–14924.2.Pearsall RS, et al. (2015) ACE‐2494, a novel GDF ligand trap, increases muscle mass upon systemic administration in mice. The 20th International Congress of the World Muscle Society.

**1-57**

**Compensatory anabolic signaling in the sarcopenia of experimental chronic arthritis**

Robert D. Little^1^, Iván Prieto‐Potin^2,3^, Sandra Pérez‐Baos^2^, Amanda Villalvilla^2^, Paula Gratal^2^, Flavia Cicuttini^1^, Raquel Largo ^2,3^ and Gabriel Herrero‐Beaumont^2,3^

^1^ *Department of Epidemiology and Preventive Medicine, School of Public Health and Preventive Medicine, Monash University, Alfred Hospital, Melbourne, Australia;* ^2^ *Bone and Joint Research Unit, Service of Rheumatology, IIS‐Fundación Jiménez Díaz, Autonomous University of Madrid, Madrid, Spain;* ^3^ *Red Temática de Investigación Cooperativa de Envejecimiento y Fragilidad (RETICEF)‐Instituto de Salud Carlos III, Madrid, Spain*

**Background and Aims:** Inflammatory activity in rheumatoid arthritis may alter the regulation of muscle mass leading to a secondary sarcopenia, commonly termed rheumatoid cachexia (RC). The mechanisms and response to muscle atrophy in RC are poorly understood. We aimed to characterize the alterations to muscle structure and the relative expression of a range of anabolic and catabolic regulatory factors.

**Methods:** Twenty male, adult rabbits were randomly assigned to antigen‐induced arthritis (AiA) or control groups. Animals were weighed weekly, and serum, tibialis anterior, gastrocnemius, and extensor digitorum longus muscles were collected. Muscle sections underwent haematoxylin and eosin staining for total cross‐sectional area and diameter, RAM11, Pax7 immunohistochemistry to identify macrophages and satellite cells, respectively, and immunofluorescence to calculate myonuclei. Gene expression of muscular IL‐1β, IL‐6, TNF, CCL‐2, myostatin, MuRF‐1, and atrogin‐1 were quantified. Protein levels of myostatin, Pax7, pSTAT1, and pSTAT3 were measured. Myostatin was also determined in synovial tissue and circulation, alongside serum C‐reactive protein (CRP).

**Results:** Antigen‐induced arthritis rabbits exhibited significantly less weight gain than controls and increase in serum CRP. AiA rabbit muscles were lighter and showed a reduction in CSD and increased number of myonuclei. Atrogin‐1 and MuRF‐1 were up‐regulated in the AiA group alongside a two‐fold increase in IL‐1β mRNA despite a decrease in CCL‐2 and a reduction in TNF and equivalent IL‐6 mRNA levels. We observed a decrease in pSTAT3, unchanged pSTAT1, and increased Pax7 levels. AiA rabbits showed reduced myostatin mRNA and protein from gastrocnemii and a decrease in synovial myostatin with a congruent reduction in serum.

**Conclusions:** Chronic arthritis induced an RC‐like secondary sarcopenia with increased muscle protein breakdown. Elevated serum CRP and muscle IL‐1β may indicate a systemic inflammatory state with a compensatory anabolic effort suggested by myonuclear expansion, increased Pax7, reduced pSTAT3, and serum, synovial, and muscular myostatin.

**1-58**

**Deciphering the pathophysiology of the tuberculosis wasting syndrome from the pathogenicity of Mycobacterium tuberculosis**

Martin M. Sampa

*Department of Animal Sciences, University of Zambia, Lusaka, Zambia*

**Background and Aims:** Tuberculosis, due to *Mycobacterium tuberculosis* infection, is the leading cause of wasting disorders worldwide. Although the pathophysiology of wasting remains unresolved, emerging evidence suggests involvement of microbial pathogenicity‐caused perturbations of the Warburg effect in M1 macrophages. This suggests a common pathway with cachexia of cancers. The aims of this study were to unravel the genetic and molecular nature of the perturbations and decipher how they initiate wasting.

**Methods:** Polya's heuristics algorithm was used. A comparison between *M.bovis* and *M.tb* unravelled the genetic basis of *M.tb* pathogenicity. The enzyme encoded by the gene located perturbation points in the Warburg effect. Cook and Campbell conditions were used to validate the results.

**Results:**

1. Rv1617 *pykA M.bovis* and *M.tb* have all genes of glycolysis in common except Rv 1617 or pykA, which is only present in *M.tb*.
2. Phosphoenolpyruvate Rv 1617 pykA encodes pyruvate kinase, which catalyses the last step of glycolysis. The presence of microbial pyruvate kinase suggests that a shunt in the glycolysis pathway is created resulting in:
  2.1 Host Pyruvate and ATP are not produced.
  2.1 Parasite Parasite produces its own pyruvate and ATP.
3. Gluconeogenesis The failure to complete Glycolysis cannot be compensated by the pentose phosphate pathway because its metabolic intermediates enter the lower part of Glycolysis. The alternative is [generating] using gluconeogenesis which generates Glucose in the liver from muscle‐derived amino acids. Initiating the catabolic process.

**Conclusions:** Glycolytic‐ATP dependent M1 macrophages are the principal locale of *M.tb* parasites. Perturbations of glycolysis induce compensatory mechanisms involving gluconeogenesis. Glucose generated via gluconeogenesis is transported to macrophages, which breaks it down via glycolysis repeating the same failed process, these are the initiatory steps of wasting.

**1-59**

**Mineralocorticoid receptor activation affects skeletal muscle homeostasis**

Alessandra Feraco, Francesca Molinari, Andrea Armani, Elisabetta Ferraro and Giuseppe Rosano

*Laboratory of Pathophysiology of Cachexia and Metabolism of Skeletal Muscle, IRCCS San Raffaele Pisana, Rome, Italy*

**Background and Aims:** It is known that mineralocorticoid receptor (MR) activation affects adipocyte function and that MR antagonism is able to improve insulin signaling in a mouse model of diet‐induced obesity. Interestingly, MR blockade affects skeletal muscle metabolism, improving insulin signaling. In addition, renin–angiotensin–aldosterone system activation leads to skeletal muscle atrophy in mice. Our aim is to evaluate a possible involvement of MR activation in regulating skeletal muscle homeostasis *in vitro* and *in vivo*.

**Methods:** We analysed the MR activation in a murine myoblast cell line (C2C12), and we started to characterize the impact of MR activity on skeletal muscle metabolic profile in obese mice.

**Results:** We first characterized the ontogenesis of MR in C2C12 in order to evaluate the expression of MR during *in vitro* differentiation. We observed an increase of MR protein expression in myotubes during differentiation. Thus, we treated 96 h myotubes with aldosterone and the MR antagonist spironolactone for 24 h, and we observed that aldosterone induced an increase in SGK1 protein phosphorylation, a well‐known target of MR activation in different tissues. In addition, aldosterone induced an increase phosphorylation of the key regulator of muscle metabolism AMPK as well as its substrate ACC. Importantly, spironolactone was able to revert these effects, suggesting that MR activation affects myofibre energy metabolism.

We also evaluated soleus metabolic profile in obese mice. As expected, we observed a reduction in GLUT4, PGC1‐a, and mtTFA protein levels in obese mice compared with lean controls. Further studies are necessary to explore the role of MR activation on skeletal muscle metabolism *in vivo*.

**Conclusions:** These data reveal a potential role of MR in modulating skeletal muscle development and metabolism, suggesting a potential application of MR antagonists to improve skeletal muscle function in several pathological conditions.

**1-60**

**The eccentric mechanotransduction and reversibility of interstitium hypertrophy: an experimental model of disuse sarcopenia of rabbit supraspinatus**

Jaroslaw Fabis ^1^, Marian Danilewicz^2^, Kryspin Niedzielski^3^, Jacek Zwierzchowski^4^, Robert Rokicki^5^ and Andrzej Bogucki^6^

^1^ *Department of Arthroscopy, Minimally Invasive Surgery and Sports Traumatology, Medical University of Łódź, Łódź, Poland;* ^2^ *FMC Private Medical Centre, Łódź, Poland;* ^3^ *Department of Pathology, Medical University of Lodz, Łódź, Poland;* ^4^ *Clinic of Orthopaedics and Traumatology, Polish Mother's Memorial Hospital Research Institute, Łódź, Poland;* ^5^ *Department of Hand Surgery, Medical University of Lodz, Łódź, Poland;* ^6^ *Department of Extrapyramidal Diseases, Medical University of Lodz, Łódź, Poland*

**Background:** An eccentric contraction occurs as part of the everyday activity of muscles. Although clinical investigations indicate that the limit of reversibility of hypertrophy of interstitium volume in case of disuse sarcopenia of rotator cuff muscles is grade 2 of fatty degeneration according to the Goutallier CT classification, little is known about the morphometric verification of these findings. Hence, it is essential to examine the influence of applied eccentric training on the reversibility of these changes in muscle structure caused by sarcopenia in case of normal neuromuscular transmission.

**Methods:** In 16 rabbits, the supraspinatus tendon was detached from the greater tubercle and infraspinatus and subscapularis, and a 12 week period of observation followed. This proved to be sufficient for grade >2 of fatty degeneration of supraspinatus development. The tendon was then reinserted in 12 animals. The animals were euthanized 24 weeks after tendon reconstruction. The sections of middle part of supraspinatus were stained (ATPse and haematoxylin reaction), and morphometric measurements were taken. The single‐fibre EMG study of neuromuscular transmission was carried out on four rabbits after 12 weeks from supraspinatus tendon detachment. The contralateral shoulders served as controls. The changes of interstitium volume were compared with the degree of hypertrophy of muscle fibre type II.

**Results:** The macroscopic inspection of the supraspinatus tendons revealed complete healing in all cases. There was 16.6% reduction of interstitium volume from 21.2%; however, the differences were statistically significant (*P* < 0.05). No statistically significant differences were found between controls and operated shoulder with respect to neuromuscular transmission (*P* < 0.05).

**Conclusion:** The growth of the interstitial tissue observed in an experimental model of disuse sarcopenia of rabbit supraspinatus is partially reversible via mechanotransduction through eccentric training. In case of normal neuromuscular transmission, the ratio of decrease of interstitium volume to 1% increase of the diameter of type II fibre is 0.63.

**1-61**

**MMPOWER: the effect of treatment with elamipretide in patients with genetically confirmed primary mitochondrial disease**

Amel Karaa ^1^, Bruce H. Cohen^2^, Amy Goldstein^4^, Jerry Vockley^4^ and Richard Haas^3^

^1^ *Massachusetts General Hospital, Boston, MA, USA;* ^2^ *Akron Children's Hospital, Akron, OH, USA;* ^3^ *UC San Diego School of Medicine, San Diego, CA, USA;* ^4^ *Children's Hospital of Pittsburgh, Pittsburgh, PA, USA*

**Introduction:** Elamipretide (ELAM; formerly referred to as Bendavia, MTP131, SS31) is a mitochondria‐targeting peptide that readily penetrates cell membranes and outer mitochondrial membranes to localize on the inner membrane where it associates with cardiolipin. In preclinical models, ELAM increased ATP production and improved exercise ability when both were reduced. ELAM restores normal energetics without reducing energy requirements.

**Methods:** Patients (*n* = 36) with genetically confirmed primary mitochondrial disease and symptoms of exercise intolerance and fatigue were enrolled in a randomized, double‐blind, placebo‐controlled, multiple ascending‐dose study to determine safety and efficacy of intravenous ELAM. Three‐sequential groups of subjects (16–65 years of age) received escalating doses of ELAM (0.01, 0.10, and 0.25 mg/kg/h infused for 2 h) once daily for 5 days. Each cohort was randomized to receive ELAM (*n* = 9) or placebo (*n* = 3). Study assessments included 6 min walk test (6MWT) distance walked, cardiopulmonary exercise test (VO_2_ max), a Daily Symptom Questionnaire, biomarkers, safety labs, vital signs, and adverse events performed at baseline and following treatment.

**Results:** Elamipretide increased the 6MWT distance in a dose‐dependent manner: 65 m increase in the highest dose‐treated subjects compared with 20 m for placebo (test for trends, *P* = 0.01). In a model adjusting for gender and baseline distance, distance change was significantly greater for the highest dose than that observed for placebo (*P* = 0.03). There were no significant differences in VO_2_ max, symptom change, biomarker levels, or adverse events.

**Conclusions:** Treatment with ELAM after 5 days resulted in a dose‐dependent increase in distance walked on the 6MWT with few adverse events, which did not differ from those seen in controls. This first dose‐finding study suggests that ELAM may reduce the extent of exercise disability in patients with primary mitochondrial disease.

**1-62**

**Elamipretide improves skeletal muscle function in elderly subjects: results from a randomized, double‐blind, placebo‐controlled, single IV dose study**

Baback Roshanravan, Z. Liu, A. S. Ali, John K. Amory, H. L. Robertson, C. Goss, Erick G. Shankland, David M. Marcinek and Kevin E. Conley

*Department of Radiology, University of Washington Medical Center, Seattle, WA, USA*

**Introduction:** Mitochondrial dysfunction leads to reduced supply of ATP and may lead to overproduction of intracellular reactive oxygen species (ROS). Excess ROS and subsequent oxidative damage to mitochondrial inner membrane structures and cardiolipin lead to sarcopenia and exercise intolerance.

The peptide elamipretide (ELAM; formerly referred to as Bendavia, MTP‐131, SS‐31) readily penetrates and localizes on the inner mitochondrial membrane where it associates with cardiolipin. Pre‐clinical studies showed improved integrity and efficiency of the electron transport chain and improvements in ATP synthesis and reduced production of ROS. ELAM improved muscle function and biomarker levels in multiple animal models of muscle dysfunction including age‐induced, chronic skeletal muscle dysfunction. The purpose of this study was to determine the effect of ELAM on skeletal muscle energetics and performance in elderly subjects.

**Methods:** Elderly subjects (*n* = 39; ≥60 and ≤85 years of age) with mitochondrial dysfunction received a single IV dose of ELAM 0.25 mg/(kg **·** h), infused over 2 h. Following infusion and on day 7, ATP max and mitochondrial coupling of ATP synthesis per O_2_ uptake (P/O) were measured. A sustained hand fatigue test determined exercise tolerance.

**Results:** A single ELAM infusion resulted in an increase in mitochondrial ATP synthesis compared with placebo (*P* = 0.055) (*Figure* 1). Compared with a previously studied similar population, the resultant increase was similar to that obtained after 6 months of exercise training (*Figure* 2). There was also a trend towards improved muscle performance vs. placebo (*Figure* 3.).

**Conclusions:** Elamipretide improved the production of skeletal muscle ATP synthesis in elderly subjects with reduced mitochondrial function. Further studies are warranted to determine whether chronic administration would reduce the incidence of sarcopenia and exercise intolerance in aging and other secondary mitochondrial diseases.

Figure 1

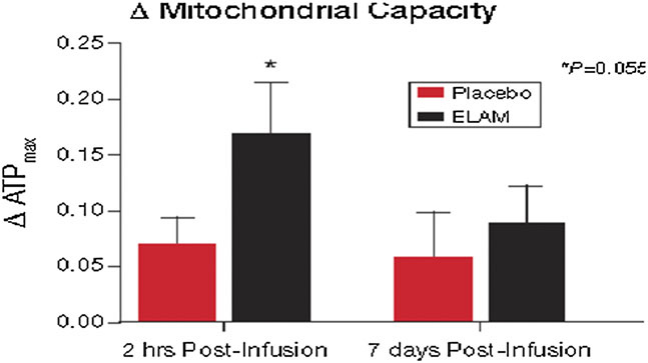

Figure 2

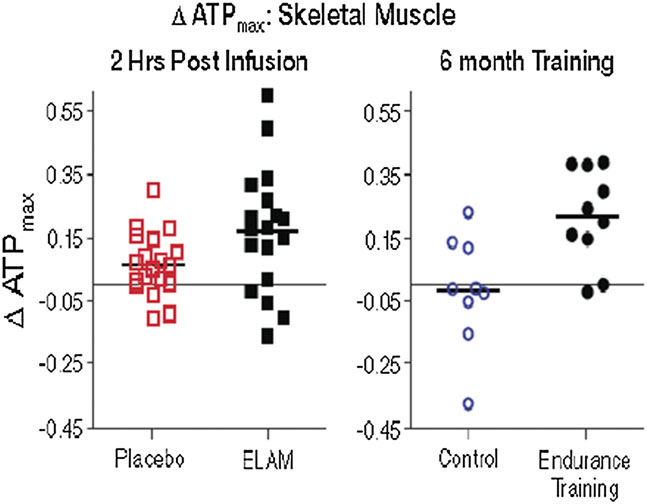

Figure 3

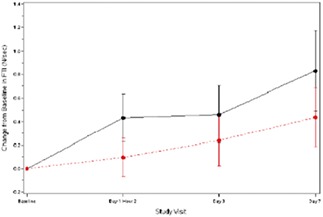

**1-63**

**The association between sleep‐disordered breathing and peripheral endothelial dysfunction in patients with acute ischemic stroke**

Nadja Scherbakov ^1,2^, Anja Sandek^3,4^, Nicole Ebner^3,4^, Miroslava Valentova^3,4^, Alexander Heinrich Nave^1^, Stephan von Haehling^3,4^, Stefan D. Anker^3,4^, Karl Georg Haeusler^1,5^ and Wolfram Doehner^1,2,6^

^1^ *Center for Stroke Research Berlin CSB, Charité – Universitätsmedizin Berlin, Berlin, Germany;* ^2^ *German Centre for Cardiovascular Research (DZHK), partner site Berlin, Berlin, Germany;* ^3^ *Innovative Clinical Trials, Department of Cardiology and Pneumology, University Medicine Goettingen (UMG), Goettingen, Germany;* ^4^ *German Centre for Cardiovascular Research (DZHK), partner site Goettingen, Goettingen, Germany;* ^5^ *Department of Neurology, Charité – Universitätsmedizin Berlin, Berlin, Germany;* ^6^ *Department of Cardiology, Charité – Universitätsmedizin Berlin, Berlin, Germany*

**Introduction** Sleep‐disordered breathing (SDB) is a highly prevalent risk factor in cardiovascular disease including stroke and has been associated with adverse outcome. Impairment of the autonomic nervous system in the acute phase of stroke may also be the cause of SDB. In the present prospective study, we aimed to investigate the effects of SDB on myocardial and endothelial function in patients with acute ischemic stroke and at 1 year follow up.

**Methods** Overall, 101 patients with acute ischemic stroke affecting the territory of the middle cerebral artery (69 ± 12 years, BMI 28.5 ± 4.6, kg/m^2^) were prospectively enrolled in 2010 and 2011. Using a Holter‐ECG device (Cardioday, Getemed, Teltow, Germany), thoracic impedance analysis was started on stroke unit admission (4 ± 2 days post‐stroke). Presence of SDB was defined by the apnea–hypopnea index ≥5 episodes per hour. All stroke patients underwent transthoracic echocardiography and peripheral endothelial function testing [using the EndoPAT 2000 (Itamar, Israel) plethysmograph]. Functional status was assessed by the modified Rankin scale (mRS) score and the Barthel index. One year after the index stroke, 41 stroke patients underwent follow‐up examinations.

**Results** While the median apnea–hypopnea index was 5.5 (IQR 1–13) episodes per hour, 56% of all stroke patients fulfilled the criteria of SDB. Compared with non‐SDB patients, SDB patients had a lower physical and functional performance (Barthel index 79 ± 26 vs. 64 ± 36, *P* = 0.023; mRS 1.8 ± 1.2 vs. 2.8 ± 1.6, *P* < 0.001). In patients with SDB peripheral endothelial function was reduced compared with patients without SDB (RHI 1.75 ± 0.33 vs. 1.98 ± 0.42, *P* < 0.01). Peripheral endothelial function was independently associated with SDB after adjustment for age, BMI, and sex [OR 0.23 (CI 0.065–0.815), *P* = 0.023]. No difference in echocardiographic parameters was observed between stroke patients with or without SDB.

In the follow‐up cohort, SDB prevalence decreased from 59% (*n* = 24) on admission to 14% (*n* = 6) (*P* < 0.001). The peripheral endothelial function improved significantly in stroke patients with former SDB but not in patients with persisting SDB (*P* = 0.034).

**Conclusions** Sleep‐disordered breathing was highly prevalent in the acute phase after ischemic stroke. Stroke severity and functional impairment were higher in patients with SDB. Peripheral endothelial dysfunction was independently associated with SDB and improved with loss of SDB.

**1-64**

**Comparison between sarcopenia and cachexia in men with chronic heart failure: results from the Studies Investigating Co‐morbidities Aggravating Heart Failure**

Amir Emami, Masakazu Saitoh, Miroslava Valentova, Anja Sandek, Ruben Evertz, Nicole Ebner, Stefan D. Anker and Stephan von Haehling

*Department of Cardiology and Pneumology, University of Goettingen Medical School, Goettingen, Germany*

**Aims**: Sarcopenia and cachexia are frequent co‐morbidities among patients with chronic heart failure (HF). As opposed to cachexia, patients with sarcopenia do not usually show weight loss but present with reduced exercise capacity and muscle strength. Both are more likely to present with advanced disease than patients with HF without wasting.^1^ We compared the prevalence and clinical impact of sarcopenia and cachexia in male patients with CHF.

**Methods**: We prospectively enrolled 218 male outpatients with chronic HF including 164 (75.2%) with HF with reduced ejection fraction and 54 (24.8%) with HF with preserved ejection fraction. We found four groups of patients in this cohort who presented with sarcopenia (sarcopenia group), cachexia (cachexia group), cachexia and sarcopenia at the same time (cachexia–sarcopenia group), and also with neither type of wasting (no wasting). The appendicular skeletal muscle mass of the arms and the legs combined was assessed by dual‐energy X‐ray absorptiometry. We analysed the muscle strength in the arms and legs, and all patients underwent a 6 min walk test (6‐MWT) and cardiopulmonary exercise testing. Sarcopenia was defined as the appendicular muscle mass 2 SD below the mean of a healthy reference group of adults aged 18–40 years as suggested for the diagnosis of sarcopenia in healthy ageing.^2^ Furthermore, cachexia was defined when there was weight loss exceeding 5% of the body weight within the previous 3–12 months combined with symptoms characteristic for cachexia (e.g. fatigue), loss of skeletal muscle, and biochemical abnormalities (e.g. anaemia or inflammation).^3^ In statistical analysis, one‐way analysis of variance and analysis of covariance were used to compare the results between the four groups. Data were adjusted for age, body mass index, and New York Heart Association classification as covariates.

**Results**: Sarcopenia alone was detected in 31 (14.2%) patients, and cachexia alone was observed in 36 (16.5%) cases. Sarcopenia and cachexia were simultaneously present in 13 (6.0%) additional patients. The cachexia–sarcopenia group had significantly lower handgrip values (33.37 ± 2.68 kg) in comparison with the cachexia group (41.8 ± 1.67 kg, *P* = 0.009) and the group without wasting (40.66 ± 0.86 kg, *P* = 0.01). Furthermore, the cachexia–sarcopenia group had significantly lower peak oxygen uptake (peak VO_2_: 13.94 ± 1.36 mL/kg/min) in comparison with the cachexia group (17.80 ± 0.75 mL/kg/min, *P* = 0.015) and the group without wasting (18.43 ± 0.39 mL/kg/min, *P* = 0.002). However, the sarcopenia group had significantly lower results for the 6‐MWT (398.7 ± 24.14 m) in comparison with the group without wasting (454.5 ± 133.2 m, *P* = 0.038). On the other hand, the 6‐MWT was not significantly lower in the cachexia–sarcopenia group. Considerably, appendicular skeletal muscle index (ASMI) is lower in the cachexia–sarcopenia group (6.97 ± 0.185 kg/m^2^) in comparison with the cachexia group (8.48 ± 0.107, *P* < 0.001) and the group without wasting (8.25 ± 0.057, *P* < 0.001).

**Conclusions**: In comparison, simultaneously cachectic and sarcopenic male patients with chronic HF seem to have the lowest handgrip and peak VO_2_, but sarcopenic patients tend to have the lowest 6‐MWT results. Muscle wasting seems to have a bigger role than cachexia in decreasing functional status among male patients with chronic HF.

References:

1. Fülster S, Tacke M, Sandek A, Ebner N, Tschöpe C, Doehner W, Anker SD, von Haehling S. Muscle wasting in patients with chronic heart failure: results from the studies investigating co‐morbidities aggravating heart failure (SICA‐HF). Eur Heart J. 2013:512‐9.

2. Morley JE, Baumgartner RN, Roubenoff R, Mayer J, Nair KS. Sarcopenia. J Lab Clin Med. 2001:231‐43.

3. von Haehling S, Anker SD. Cachexia as a major underestimated and unmet medical need: facts and numbers. J Cachexia Sarcopenia Muscle. 2010:1‐5.

**2-14**

**Cachexia‐associated transforming growth factor‐β superfamily ligands as circulating biomarkers of pulmonary hypertension**

Elisabetta Ferraro, Laura Vitiello, Roberto Badagliacca, Maurizio Volterrani, Roberto Poscia, Giuseppe Rosano and Dario Vizza

*Laboratory of Pathophysiology of Cachexia and Metabolism of Skeletal Muscle, IRCCS San Raffaele Pisana, Rome, Italy*

**Background and Aims:** Pulmonary arterial hypertension (PAH) is characterized by progressive elevation of pulmonary arterial pressure due to high muscularization of pulmunary arteries whose lumen and elasticity decrease. This leads to progressive right‐sided heart failure and to a high mortality rate. Although the molecular mechanisms underlying PAH are not clearly understood, this disease has been associated with mutations in several genes mostly belonging to the transforming growth factor‐β superfamily. In addition to genetic determinants as diagnostic tools, the availability of optimal circulating biomarkers is critical in clinical practice for an accurate assessment of prognosis and treatment responses. Here, we sought to identify new circulating biomarkers of PAH and to evaluate the impact of specific therapy on their concentration.

**Methods:** The concentration of 17 molecules (Troponin‐T, NT‐proBNP, TNF‐α, IL17a, IL6, IL‐1Ra, IL‐1β, CRP, GDF‐15, activin‐A, GDF‐8, BMP‐9, BMP‐4, endothelin‐1, tenascin‐C, VEGF and PIM‐1) was assessed in the serum of 20 age‐matched and gender‐matched controls and 60 PAH patients before and after specific therapy. We used standard single immune assays and the Luminex technology in order to evaluate the levels of several molecules by using small volumes of serum.

**Results:** In our analysis, we confirmed that several inflammatory markers increase in PAH patients along with the levels of endothelin‐1 and of some transforming growth factor‐β superfamily ligands, which have recently been proposed also as cachexia biomarkers in other syndromes.

**Conclusions:** Because a single biomarker for PAH is unlikely to meet all requirements, our data suggest the possible use of some ActRII ligands as prognostic biomarkers, in addition to previously identified ones. Moreover, the possible association with a cachectic phenotype might suggest new additional therapeutic interventions for PAH, which should target the skeletal muscle in order to protect it from atrophy and allow a better quality of life and, possibly, prognosis.

**2-15**

**Targeted medical nutrition for cachexia in chronic obstructive pulmonary disease (COPD): a randomized, double‐blind controlled trial**

Philip C. Calder^1^, Alessandro Laviano^2^, Fredrik Lonnqvist^3^, Maurizio Muscaritoli^4^, Maria Öhlander^5^ and Annemie Schols ^6^

^1^ *Human Development and Health Academic Unit, Faculty of Medicine, University of Southampton, Southampton, UK;* ^2^ *NIHR Southampton Biomedical Research Centre, University Hospital Southampton NHS Foundation Trust and University of Southampton, Southampton, UK;* ^3^ *Department of Clinical Medicine, Sapienza University of Rome, Rome, Italy;* ^4^ *Department of Molecular Medicine and Surgery and the Center for Molecular Medicine, Karolinska Institute, Stockholm, Sweden;* ^5^ *Smartfish AS, Oslo, Norway;* ^6^ *Department of Respiratory Medicine, NUTRIM School of Nutrition and Translational Research in Metabolism, Maastricht University Medical Centre, Maastricht, Netherlands*

**Objective**: Cachectic patients with COPD may benefit from nutritional supplementation. This double‐blind, randomized, controlled trial evaluated the safety and efficacy of targeted medical nutrition (TMN) vs. an isocaloric comparator in (pre‐)cachectic patients with COPD.

**Methods**: Patients ≥50 years of age with moderate‐to‐severe COPD and involuntary weight loss or low BMI (16–18 kg/m^2^) were randomized 1 : 1 to receive TMN (Smartfish Remune, ~230 kcal; 2 g omega‐3 fatty acids; 10 g 25‐hydroxy‐vitamin D3) or an isocaloric comparator twice daily for 12 weeks (ClinicalTrials.gov Identifier: NCT02442908). Primary safety endpoints comprised adverse events and changes in vital signs, laboratory parameters, and concomitant medications. Secondary efficacy endpoints included changes in weight, exercise tolerance, and systemic inflammation.

**Results**: Forty‐five patients with similar distribution between men and women were randomized to receive TMN (*n* = 22; mean 69.2 years) or isocaloric comparator (*n* = 23; mean 69.7 years). TMN was well tolerated, and compliance was good. Both groups gained weight, but the TMN group gained comparatively more fat mass (*P* = 0.001). Significant improvements in systolic blood pressure (*P* = 0.04), increase in HDL (*P* = 0.03), decrease in triglycerides (*P* = 0.02), and reduced BORG score for fatigue (*P* = 0.02) and dyspnoea (*P* = 0.04) during the 6 min walk test were observed in the TMN vs. comparator group by week 12.

**Conclusion**: A TMN containing high‐dose omega‐3 fatty acids, vitamin D, and high‐quality protein shows positive effects on the metabolic profile and exercise‐induced fatigue and could therefore be beneficial for (pre‐)cachectic patients with COPD.

**3-12**

**Use of routinely available clinical, nutritional, and functional criteria to classify cachexia in advanced cancer patients**

Antonio A. L. Vigano ^1,2^, José A. Morais^3^, Lorella Ciutto^1,4,5^, Leonard Rosenthall^6^, Jonathan Di Tomasso^4^, Sarah Khan^1^, Henry Olders^1^, Manuel Borod^2^ and Robert D. Kilgour^1,7^

^1^ *McGill Nutrition and Performance Laboratory (MNUPAL), Quebec, Canada;* ^2^ *Supportive and Palliative Care, McGill University Health Centre, Quebec, Canada;* ^3^ *Geriatric Medicine, McGill University Health Centre, Royal Victoria Hospital, Quebec, Canada;* ^4^ *School of Dietetics and Human Nutrition, McGill University, Quebec, Canada;* ^5^ *Centre Hospitalier Universitaire Vaudois, Service d'endocrinologie, Diabétologie et Métabolisme, Nutrition Clinique, Lausanne, Switzerland;* ^6^ *Department of Radiology, McGill University Health Centre, Quebec, Canada;* ^7^ *Department of Exercise Science, Concordia University, Quebec, Canada*

**Background:** Cachexia is a highly prevalent syndrome in cancer and chronic diseases. However, because of the heterogeneous features of cancer cachexia, its identification and classification challenge clinical practitioners.

**Objective:** We sought to determine the clinical relevance of a cancer cachexia classification system in advanced cancer patients.

**Design:** Beginning with the four‐stage classification system proposed for cachexia [non‐cachexia (NCa), pre‐cachexia (PCa), cachexia (Ca), and refractory cachexia (RCa)], we allocated patients in the cachexia stages according to five classification criteria available in clinical practice: (i) biochemistry (high C‐reactive protein or leukocytes, or hypoalbuminemia, or anaemia); (ii) food intake (normal/decreased), weight loss; (iii) moderate performance status (≤5%); (iv) significant performance status (>5%/past six months); and (v) performance status (Eastern Cooperative Oncology Group Performance Status ≥3). Thereafter, we determined if symptoms severity, body composition changes, functional levels, hospitalization, and survival rates varied significantly across patients according to the cachexia stages.

**Results:** Two hundred and ninety‐seven advanced cancer patients with primary gastrointestinal and lung tumours were included. Patients were classified into Ca (36%), PCa and RCa (21%, respectively), and NCa (15%). Significant (*P* < 0.05) differences were observed among the cachexia stages for most of the outcomes (symptoms, body composition, handgrip strength, emergency room visits, and length of hospital stays) according to the severity of cachexia. Survival analysis showed differences among all stages except between PCa and Ca.

**Conclusion:** The five criteria that we tested in this study can be used to identify the cachexia stages and predict important clinical, nutritional, and functional outcomes. The lack of statistical difference between PCa and Ca in all the clinical outcomes examined may suggest either that the PCa group includes patients already affected by early cachexia or that more precise criteria need to be used to differentiate PCa vs. Ca patients. More studies are required to validate these findings.

**3-13**

**The relationship between positron emission tomography (PET) / computed tomography (CT)‐derived measurements of muscle glucose metabolism and myofibrillar protein synthesis in oesophageal cancer patients**

Michael Ramage ^1^, Alisdair J. MacDonald^1^, Dilip Patel^2^, D. A. Christopher Deans^1^, James A. Ross^3^, Tom Preston^4^, Richard J. E. Skipworth^1^ and Ken C. H. Fearon*^1^

^1^ *Clinical Surgery, Royal Infirmary of Edinburgh, Edinburgh, UK;* ^2^ *Radiology, Royal Infirmary of Edinburgh, Edinburgh, UK;* ^3^ *Tissue Injury and Repair Group, University of Edinburgh, Edinburgh, UK;* ^4^ *Stable Isotope Research Laboratory, Scottish Universities Research Centre, Glasgow, UK*

**Introduction:** We have shown previously that habitual myofibrillar muscle protein synthesis is normal in patients with upper gastrointestinal cancer. In such patients, ^18^F‐FDG PET/CT, which assesses glucose uptake within cancer cells, is a standard staging investigation. However, PET/CT has not been used to assess the glucose metabolism of muscle in cancer patients. We aimed to investigate the relationship between PET/CT‐derived skeletal muscle standardized uptake values (SUV) as a measure of muscle glucose metabolism and myofibrillar protein synthetic rates.

**Methods:** ^18^F‐FDG PET/CT scans were performed pre‐operatively on oesophageal cancer patients (*n* = 9; M : F 5 : 4). SUV measurements at the L3 level, and of liver, were collected using automated CT‐PET viewing software. Deuterium‐labelled water was administered, and serum samples were taken over the subsequent week followed by a quadriceps muscle biopsy. Rectus biopsies were taken intra‐operatively. Deuterium enrichment was measured in body water, serum alanine, and alanine in the myofibrillar component of muscle using gas chromatography–pyrolysis–isotope ratio mass spectrometry.

**Results:** Median time between PET/CT and muscle biopsy was 76 days (range 17–98 days). Muscle SUV values showed significant variation between muscle groups (rectus abdominis, quadratus lumborum, erector spinae, obliques, and psoas major), muscle laterality (right vs. left), and individual patients. Standardization of muscle SUV to background liver SUV (SUVL) reduced much of the variability. Rectus abdominis SUVL correlated with absolute synthetic rate (ASR‐g/day) in rectus abdominis (*r* = 0.833, *P* = 0.005; Spearman's correlation) and quadriceps femoris (*r* = 0.717, *P* = 0.03).

**Conclusion:** Despite the length of time between PET/CT and muscle biopsies and the presence of other confounders (such as neoadjuvant chemotherapy), this exploratory analysis suggests that glucose metabolism and myofibrillar protein synthesis may be related in the skeletal muscle of oesophageal cancer patients. Although there is considerable variability in muscle SUV, standardization of SUV in relation to host liver might allow inter‐individual comparisons.

**3-14**

**Effects of activin receptor ligand blocking on survival and muscle physiology in C26 colon cancer cachexia**

Tuuli A. Nissinen ^1^, Jaakko Hentilä^1^, Fabio Penna^2^, Juulia Lautaoja^1^, Mika Silvennoinen^1^, Tanja Holopainen^3^, Arja Pasternack^4^, Olli Ritvos^4^, Riikka Kivelä^3^ and Juha J. Hulmi^1,4,5^

^1^ *Department of Biology of Physical Activity, Neuromuscular Research Center, University of Jyväskylä, Jyväskylä, Finland;* ^2^ *Department of Clinical and Biological Sciences, University of Turin, Turin, Italy;* ^3^ *Interuniversity Institute of Myology, Italy;* ^4^ *Wihuri Research Institute and Translational Cancer Biology Research Program, University of Helsinki, Helsinki, Finland;* ^5^ *Department of Physiology, Faculty of Medicine, University of Helsinki, Helsinki, Finland*

**Background and Aims.** Prevention of cachexia by blocking activin receptor ligands may improve survival. The aim of this study was to investigate the effects of increasing muscle mass by soluble activin receptor (sACVR2B‐Fc) only before or both before and after the onset of C26 cancer cachexia.

**Methods.** BALB/c mice were subcutaneously inoculated with C26 cancer cells or vehicle control. Tumour‐bearing mice were randomized into three groups: (i) placebo treatment; (ii) sACVR2B‐Fc (produced in house) treatment only before tumour formation based on our pilot experiments; and (iii) sACVR2B‐Fc treatment before and after tumour formation throughout the experiment. For investigation of survival, mice were followed until the humane endpoint criteria were fulfilled. The Cox regression analysis revealed that body weight change from day 10 to 11 after cancer cell inoculation predicted survival. Thus, for investigation of the potential mechanisms underlying the differences in survival time, another experiment for the same groups was performed with a pre‐determined endpoint at day 11 after cancer cell inoculation. Statistical significance was set at *P* < 0.05.

**Results.** C26 cancer resulted in cachexia manifested by significant loss of muscle and fat mass. sACVR2B‐Fc treatment prevented cancer‐induced muscle, but not fat loss. Survival was improved only in mice treated continuously with sACVR2B‐Fc. Tumour‐bearing mice had decreased muscle protein synthesis in tibialis anterior and diaphragm muscles. Also, the amount of ubiquitinated proteins increased in tumour‐bearing mice, suggesting increased proteolysis. In this terminal stage of cancer, sACVR2B‐Fc treatment had only minor effects on these physiological parameters.

**Conclusions.** These results suggest that prevention of muscle wasting by blocking activin receptor ligands improves survival in C26 cachexia. However, increasing muscle mass only prior to cachexia may not be enough to improve survival if muscle mass is not maintained. *This work was supported by Academy of Finland grant No. 275922.*

**3-15**

**Effects of activin receptor ligand blocking and C26 tumour‐induced cancer cachexia on endoplasmic reticulum stress and unfolded protein response in skeletal muscle**

Jaakko Hentilä ^1^, Tuuli A. Nissinen^1^, Ayhan Korkmaz^2^, Sanna Lensu^1^, Arja Pasternack^3^, Olli Ritvos^3^, Mustafa Atalay^2^ and Juha J. Hulmi^1,3^

^1^ *Department of Biology of Physical Activity, Neuromuscular Research Center, University of Jyväskylä, Jyväskylä, Finland;* ^2^ *Institute of Biomedicine, Physiology, University of Eastern Finland, Kuopio, Finland;* ^3^ *Department of Physiology, Faculty of medicine, University of Helsinki, Helsinki, Finland*

**Background and Aims**. Accumulation of misfolded proteins into endoplasmic reticulum (ER) leads to ER stress, which is resolved by unfolded protein response (UPR). Little is known about the C26 tumour‐induced cachexia or activin/myostatin blocking on ER stress and UPR in skeletal muscle. The first aim was to elucidate the acute and 2 week effects of activin receptor ligand blocking on ER stress/UPR and protein carbonyls in healthy skeletal muscle. The second aim was to elucidate the effects of C26 cancer‐induced cachexia on ER stress/UPR and protein carbonyls with or without activin receptor ligand blocking in skeletal muscle.

**Methods.** Mice were sacrificed either 1 or 2 days after a single sACVR2B‐Fc (produced in house) administration or after 2 weeks of bi‐weekly administration. For the cancer experiment, C26 cells were inoculated to mice, and sACVR2B‐Fc or PBS placebo was administered before C26 inoculation or before and after C26. The mice were sacrificed 11 days after the C26 inoculation.

**Results:** Single sACVR2B‐Fc administration had no effect on ER stress/UPR 1 or 2 days after the administration, but it increased protein carbonyls (*P* < 0.05). Two‐week sACVR2B‐Fc administration increased some (GRP78, p‐eIF2α at ser51, HSP47) of the ER stress/UPR markers, and protein carbonyls were slightly, but significantly, elevated. C26 cancer‐induced cachexia decreased some (p‐eIF2α at ser51, HSP47) of the ER stress/UPR markers but had no effect on protein carbonyls. sACVR2B‐Fc administration had no effect on ER stress/UPR or protein carbonyls in C26 cancer cachexia.

**Conclusions**. sACVR2B‐Fc administration does not acutely result in ER stress but 2‐week administration may increase ER stress/UPR and may increase oxidative stress acutely and in short term. ER stress/UPR is unchanged or even slightly decreased in skeletal muscle 11 days after C26 inoculation and at this phase of cachexia sACVR2B‐Fc administration has no effect on ER‐stress/UPR. *This work was supported by Academy of Finland grant nos 137787 and 275922.*

**3-16**

**An evaluation of cachexia prevalence and patient‐perceived need of clinical attention to weight loss and nutrition in a general cancer population**

Ola Magne Vagnildhaug ^1,2^, Sigrun Saur Almberg^1,2^, Morten Thronæs^1,2^, Anne Kari Knudsen^1,3^, Cinzia Brunelli^1,4^, Stein Kaasa^1,3^, Barry Laird^1,5,6^ and Tora Skeidsvoll Solheim^1,2^

^1^ *European Palliative Care Research Centre (PRC), Department of Cancer Research and Molecular Medicine, Faculty of Medicine, NTNU – Norwegian University of Science and Technology, Trondheim, Norway;* ^2^ *Cancer Clinic, St. Olavs Hospital, Trondheim University Hospital, Trondheim, Norway;* ^3^ *Department of Oncology, Oslo University Hospital, University of Oslo, Oslo, Norway;* ^4^ *Palliative Care, Pain Therapy and Rehabilitation Unit, Fondazione IRCCS Istituto Nazionale dei Tumori, Milano, Italy;* ^5^ *Edinburgh Cancer Research UK Centre, University of Edinburgh, Edinburgh, UK;* ^6^ *Beatson West of Scotland Cancer Centre, Glasgow, UK*

**Background:** There is considerable variance in prevalence estimates of cancer cachexia because of the heterogeneity of cachexia definitions. Lack of knowledge about cachexia among healthcare professionals has led to under‐recognition and avoidance of the topic when faced with the patient. The objectives of this study are to provide an accurate estimate of cachexia prevalence in a general cancer population, to assess patient‐perceived needs of attention to weight loss and nutrition, and to explore which factors signal such a need.

**Methods:** A cross‐sectional study was undertaken in adult inpatients and outpatients with cancer at three different hospitals in Central Norway. Trained personnel reported on disease‐specific items, while patients self‐reported demographic data, weight, symptom intensity scores, and need of healthcare personnel's attention to weight loss and nutrition. Cachexia was defined as (i) weight loss >5% or (ii) weight loss >2% and BMI <20 kg/m^2^. Prevalence of cachexia as well as patient‐perceived need of clinical attention to cachexia was estimated. Linear regression was used to explore which factors were associated with such a need.

**Results:** Data were available on 386 of 426 eligible patients. Median age (IQR) was 65 years (56–72), 214 (55%) were male, and 302(78%) had a performance status of 0–1 (ECOG). Prevalence of cachexia (inpatients/outpatients) was 51%/22%. Prevalence was highest in patients with gastrointestinal cancer (61%/42%) and lung cancer (83%/38%). There was no major difference in prevalence between patients with metastatic disease (55%/24%) and localized disease (47%/19%). Moreover, 20% of inpatients and 15% of outpatients said they wanted more attention to weight loss and nutrition. Weight loss (*P* < 0.001), depression and anxiety symptoms (*P* < 0.001), and male gender (*P* = 0.004) were independent factors associated with an increased need of attention.

**Conclusion:** Cachexia is a prevalent condition, affecting both patients with localized and metastatic cancer. Clinical attention to the condition is a sizeable unmet need.

**3-17**

**Convergence and divergence of anorexia and cancer cachexia**

McKenna Williams^1^, Blake Woodside^2^, Ruth Patterson^1^, Katherine Ann Halmi^3^ and Pei‐an (Betty) Shih^1^

^1^ *University of California, San Diego, CA, USA;* ^2^ *University of Toronto, Toronto, Canada;* ^3^ *Cornell University, Ithaca, NY, USA*

**Background**: Anorexia nervosa and cancer cachexia are both severely debilitating, with high incidences of morbidity and mortality. Interestingly, while they have distinct and separate etiologies, they share strikingly similar magnitude of weight loss as disease progresses. In this literature review, we aim to present the convergence and divergence of anorexia nervosa, a major psychiatric illness characterized by a progressive ‘wasting phenotype’ and aberrant metabolism, and cancer cachexia, a ‘wasting syndrome’ commonly observed in late stage cancer patients that is characterized by catabolic state and skeletal muscle wasting. Examining these two disorders in parallel provides a more comprehensive understanding of their unique and shared characteristics and builds a foundation of knowledge that can ground new, testable hypotheses for further research and ultimately advance treatment strategies.

**Methods**: A systematic literature search was conducted using the MEDLINE/PubMed database. A total of 88 English language, peer‐reviewed articles were selected for analysis based on their relevance to three pre‐selected domains: body composition, inflammation, and treatment. The following keywords were employed to target articles relating to the aforementioned domains: cachexia, anorexia nervosa, anorexia–cachexia, inflammation, cytokines, nutrition, treatment, weight loss, fat mass, lean body mass, mortality, and BMI.

**Results**: A total of 88 studies were selected for analysis. In studies involving patients with anorexia nervosa (*n* = 54), the mean age was 19.81 ± 4.75 years (SD), and the mean BMI upon admission to a treatment programme was 15.34 ± 2.11 (SD). In comparison, patients with cancer cachexia had a mean age of 64.9 ± 4.66 years and mean BMI of 22.66 ± 2.22 (*n* = 2299). Anorexia nervosa results in loss of fat mass often with the retention of lean mass and muscle strength due in part to patients' excessive exercising (observed in up to 80% of patients), while cancer cachexia leads to skeletal muscle wasting resulting in loss of both fat and lean body mass alongside the loss of muscle strength. While the two diseases share ‘anorexia’, a loss of appetite for food, this loss is driven by a behaviourally self‐induced starvation in patients with anorexia nervosa in comparison with the tumoural‐associated or treatment‐induced loss of appetite in patients with cancer cachexia. Beyond the phenotypic and behavioural domains, aberrant inflammatory markers, specifically proinflammatory cytokines including TNF‐a, IL‐6, and IL‐1B, were associated with both disorders. Surprisingly, the pattern of inflammatory marker associations in AN was varied across studies. Out of 10 studies selected as relating to inflammation in anorexia nervosa, five studies reported higher circulating levels of TNF‐a or IL‐6. Three studies reported no difference in circulating levels of TNF‐a, IL‐6, or IL‐1B between patients with anorexia nervosa and a healthy control group, while two studies reported lower production levels of TNF‐a, IL‐6, or IL‐1B. One study reported lower production levels of TNF‐a and IL‐6 but higher production levels of IL‐1B. In contrast, a higher level of IL‐6 is consistently observed in cancer cachexia, resulting in it being recommended as a potential diagnostic criteria for cancer cachexia. Other inflammation markers including C‐reactive protein, TNF‐a, and IL‐1 have also been established as hallmark features of the cancer cachexia syndrome. While both anorexia nervosa and cancer cachexia can lead to death, up to 20% of anorexia nervosa patients die if untreated, whereas 20% of cancer death is directly attributed to cachexia. No effective pharmacological treatment is available for either of these disorders.

**Conclusions**: The relatively high prevalence and mortality rates of anorexia nervosa and cancer cachexia urgently demand more studies to improve our understanding of the underlying mechanisms of each disorder. Because of the anosognosic nature of anorexia nervosa and a lack of effective diagnostic biomarker for cancer cachexia, both disorders are often underdiagnosed and undertreated until the disorder progresses to an advanced state. Anorexia nervosa and cancer cachexia share evidence of aberrant systemic inflammation, yet there is a degree of contradicting evidence regarding the presentation of inflammatory markers in anorexia nervosa. Given the significant convergence and divergence of these two wasting disorders, we propose that a comparative study utilizing the state of the art multi‐omics technology may pave the way to enable a systematic investigation to better understand the similarity and differences in molecular mechanisms between these two forms of wasting disorders. These comparative studies in turn will enable the development of more sensitive and specific biomarkers for each disorder and pave the foundation needed for future research to advance treatment development for patients.

**3-18**

**Cancer cachexia and comorbidity**

Francesco Matozzo, Daniela Martinelli, Paolo Cordioli, Elena Mariani, Alessia Modè, Antonietta Perronel, Claves Reggiani, Anna Rossi, Elena Spiritelli and Laura Rigotti

*Department of Palliative Care, “Carlo Poma” Hospital, Mantova, Italy*

**Background and Aims**: Although the malignant tumours occur at all age, they affect mainly the elderly. Similarly, the most epidemiological studies indicate an exponential increase of dementia Alzheimer type (DAT) and Parkinson disease (PD) after 70 years. The cancer is characterized by an unlimited cellular proliferation, while the neurodegeneration is a process of premature cell death: in this sense, the diseases appear to be the opposing ends of the same spectrum, sharing many genes and biological pathways regulated, however, in different directions. Many studies give credence to the idea that a history of cancer might decrease the risk of neurodegenerative disease, and vice versa. We evaluated if the cancer cachexia in older patients was worsened also by chronic neurodegenerative states.

**Methods:** We conducted a retrospective observational study of 1.081 patients, 608 males and 473 females with age equal or older 70 years; all patients with advanced cancer were assisted by Palliative Home Care Service. The most common cancer types were lung 20.7%, colon and rectal 12.2%, gastric 6.9.%, breast 6.6%, pancreatic 6.6%, and prostate 5.6%. The median survival was 23 days. The presence or absence of DAT and PD was evaluated with careful clinical–anamnestic investigation, ruling out the patients with extrapiramidal disorders and neurocognitive disturbs appeared after diagnosis of cancer and chemotherapy or those with an incomplete medical history. We calculated age‐specific prevalence comparing the data with those reported in the literature:

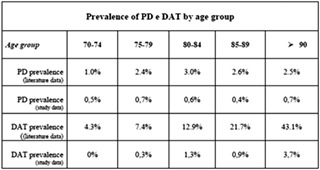

**Results and Conclusion**: As a result, the prevalence of DAT and PD was heavily reduced in all age‐specific subgroup of patients with cancer cachexia, supporting the evidence of a true inverse comorbidity between cancer and the most common age‐related neurodegenerative disease. Conversely, the cancer cachexia was associated with other diseases that share chronic inflammation: the heart disease and hypertension were the most frequently manifested (52.6%), followed by the type 2 diabetes present in 16.5% (in half of cases associated with cardiovascular disease), while the type 1 diabetes was detected only in 3.6% of cases always in association with cardiovascular disease; the chronic respiratory diseases were present in 6% like the chronic arthropathies; finally, the renal diseases were present in 4% like stroke.

References

1. Driver JA. *Inverse association between cancer and neurodegenerative disease: a review of the epidemiologic and biological evidence*. Biogerontology 2014;15:547‐557.

2. Manabe I. *Chronic inflammation links cardiovascular, metabolic and renal diseases*. Circ J 2011;75:2739‐2748.

3. Musicco M, Adorni F, Di Santo S, et al. A. *Inverse occurrence of cancer and Alzheimer disease. A population‐based incidence study.* Neurology 2013;81:1‐7.

**3-19**

**The quest for a novel biomarker for cancer cachexia: rational and design for a case control study**

Ayman Aboda ^1^, Wafa Taha^2^, Iman Attia^2^, Adel Elkady^3^, Mohamed Hegazy^2^, Mohamed Wadod^2^, Mamdouh Mostafa^2^, Mohammadreza Mohebbi^4^, Paul Lewandowski^1^, Rupinder Kaur Kanwar^1^ and Jagat Rakesh Kanwar^1^

^1^ *School of Medicine, Faculty of Health, Centre for Molecular and Medical Research (C‐MMR), Deakin University, Geelong, Australia;* ^2^ *National Cancer Institute, Cairo University, Cairo, Egypt;* ^3^ *Police Force Hospital, Giza, Egypt;* ^4^ *Biostatistics Unit, Faculty of Health, Deakin University, Geelong, Australia*

**Background:** Cachexia is the main cause of death in cancer patients. There is an urgent need for validated biomarkers that could help in the early diagnosis at the pre‐cachexia stage before sever loss of subcutaneous fat and skeletal muscle occur. It is currently not possible to conclude firmly on a specific biomarker of cancer cachexia; however, C‐reactive protein (CRP) and interleukin‐6 (IL‐6) are emerging as potential candidates of diagnosis, especially after the development of anti‐IL‐6 antibodies for the treatment for cachexia.

**Method/Design:** The proposed study will be a case control study, with one cachexia patient (*n* = 107) per two controls (*n* = 214). The primary aim of this study is to compare circulating levels of IL‐6 and CRP in patients with cancer cachexia and patients with the same cancer type who do not have cachexia. The secondary aim is to determine if IL‐6 and/or CRP could be used as a predictive biomarker for the likely onset of cancer cachexia.

**Discussion**: The drive to discover a therapy to combat cancer cachexia has become paramount, given that current strategies are generally without effect in patients with cancers who present with cachexia. Finding a cure for cachexia will impact cancer patients' worldwide, improving quality of life and potentially increasing survival in response to standard care. Achieving this goal will depend on our ability to better diagnose cachexia in cancer patients, to increase our understanding of the mechanisms that underlie wasting of adipose and muscle tissues, to identify and translate therapeutic targets to the clinic. This will standardize outcome measures to facilitate and accelerate clinical trials for improved benefit in cancer patients and their palliative care.

**3-20**

**Novel biomarker for early diagnosis of cancer cachexia**

Ayman Aboda ^1^, Wafa Taha^2^, Iman Attia^2^, Nelly Alieldin^2^, Adel Elkady^3^, Rupinder Kaur Kanwar^1^ and Jagat Rakesh Kanwar^1^

^1^ *School of Medicine, Faculty of Health, Centre for Molecular and Medical Research (C‐MMR), Deakin University, Geelong, Australia;* ^2^ *National Cancer Institute, Cairo University, Cairo, Egypt;* ^3^ *Police Force Hospital, Giza, Egypt*

**Background:** Cachexia is the main cause of death in cancer patients. The optimal therapy for cancer cachexia depends on our ability to better diagnose cachexia in cancer patients as early as possible and to understand the mechanisms responsible about cachexia. So there is an urgent need for validated biomarkers that could help in the early diagnosis at the pre‐cachexia stage before sever loss of subcutaneous fat and skeletal muscle occur.

**Method/Design:** A case control study, hospital‐based, was conducted in 357 participants, ages 18–75 years. The primary aim of the study was to compare circulating levels of IL‐6 and CRP in patients with cancer cachexia and patients with the same cancer type who do not have cachexia. The secondary aim was to determine if IL‐6 and/or CRP could be used as a predictive biomarker for the likely onset of cancer cachexia. Data from this study were also used to check if the Glasgow Prognostic Score (albumin <35 g/L = 1 and CRP >10 mg/L = 1, combined to form a prognostic score of 0 normal and 1 or 2 abnormal) could be used as a tool for diagnosis of cancer cachexia.

**Results:** Both study groups were matched regarding age and sex, and distribution of diagnosis was not significantly different in both groups; *P* value was not significant. There was a significant difference in both IL‐6 and CRP levels between cases and controls: *P* value was < 0.001 for both. There was also a significant difference in testosterone level in men between cases and controls: *P* value was < 0.001.

**Conclusion:** IL‐6 and CRP could be used as biomarkers for early diagnosis of cancer cachexia. Testosterone could be used in male patients as a biomarker for diagnosis of cancer cachexia but needs further studies with large number of participants. The Glasgow Prognostic Score could be used as a tool for early diagnosis of cancer cachexia.

**3-21**

**Does Lactoferrin have a role in cancer cachexia?**

Ayman Aboda^1^, Wafa Taha^2^, Iman Attia^2^, Nelly Alieldin^2^, Mahmoud Hassan^2^, Rupinder Kanwar^1^ and Jagat Kanwar ^1^

^1^ *School of Medicine, Faculty of Health, Centre for Molecular and Medical Research (C‐MMR), Deakin University, Geelong, Australia;* ^2^ *National Cancer Institute, Cairo University, Cairo, Egypt*

**Background:** Cancer cachexia is a multifactorial syndrome characterized by an ongoing loss of skeletal muscle mass that cannot be completely reversed by nutritional support. Finding a cure for cachexia will impact cancer patients' worldwide, improving quality of life and potentially increasing survival in response to standard care.

**Method/Design:** A retrospective longitudinal cohort study was conducted to compare the serum level of human lactoferrin and serum iron in cancer cachexia patients with that in patients with the same cancer type but do not have cachexia. Clinical audit data were collected for 87 cases, 63.2% were female and 36.8% were male and 87 controls, 58.6% were female and 41.4% were male in 2016 from outpatients who attended the National Cancer Institute in Cairo, Egypt.

**Results:** Both study groups were matched regarding age and sex, and distribution of diagnosis were not significantly different in both groups, *P* value = 0.96. Lactoferrin and serum iron were significantly lower in cases as compared with controls, *P* value = 0.002 for Lactoferrin and ≤0.001 for serum iron. Furthermore, liver functions were significantly higher in cases as compared with controls. Lactoferrin and serum iron level were negatively associated with change in BMI, *P* value is <0.001, and there is a correlation between drop in serum level of lactoferrin and changes in BMI. On the other‐hand, overall survival was not related to either serum lactoferrin or serum iron in both groups combined.

**Conclusion:** It would be beneficial to investigate if bovine lactoferrin could be used for treatment of cancer cachexia patients as bovine lactoferrin increase level of serum human lactoferrin so improve the immunity.

**4-04**

**Lipolytic activity of nodal mesenteric but not epididymal fat tissue during rat adjuvant arthritis: a model of cachectic rheumatoid arthritis**

Andrea Stofkova ^1^, Katarina Krskova^2^, Alexander Kiss^2^, Jan Liska^2^, Simon Vaculin^3^ and Jana Jurcovicova^1,2^

^1^ *Department of Normal, Pathological, and Clinical Physiology, Third Faculty of Medicine, Charles University, Prague, Czech Republic;* ^2^ *Institute of Experimental Endocrinology, Biomedical Centre, Slovak Academy of Sciences, Bratislava, Slovakia;* ^3^ *Institute of Histology and Embryology, Medical Faculty of Comenius University, Bratislava, Slovakia*

Cachectic rheumatoid arthritis, the less frequent form of the disease, is associated with loss of fat mass and often more severe course of the disease. Its experimental model is rat adjuvant arthritis (AA). Because individual fat depots display different lipolytic activities, in this model we studied the activities of adipose triglyceride lipase hormone‐sensitive lipase (HSL) and its active phosphorylated form (pHSL), in nodeless epididymal fat (eWAT) and perinodal mesenteric fat (mWAT), as well as hormones and cytokines involved in lipolysis in plasma and/or isolated adipocytes from both WATs.

Adjuvant arthritis was induced to male Lewis rats by complete Freund's adjuvant. On day 18 of maximum arthritic score, the experiment was terminated after a 12 h fast. In AA rats, we found loss of both gastrocnemius and soleus muscle mass, lowered adiposity in mWAT but not eWAT against controls. Adipose triglyceride lipase was unchanged in both WATs. HSL, pHSL, and pHSL/HSL ratio were unchanged in eWAT but enhanced in mWAT. Immunohistochemical staining confirmed enhanced presence of pHSL in mWAT adipocytes of arthritic rats. Plasma norepinephrine and epinephrine were enhanced in AA group; in eWAT adipocytes, norepinephrine was enhanced against controls, while in mWAT adipocytes, epinephrine was higher against controls and also over eWAT of AA rats. Adipocyte leptin was depleted in arthritic animals in accordance with body weight loss. Cytokine‐induced neutrophil chemoattractant‐1 (CINC‐1/CXCL1), monocyte chemoattractant protein‐1 (MCP‐1/CCL2), and IL‐1β, IL‐6, and IL‐10 concentrations were not enhanced in isolated adipocytes. Our results clearly show presence of lipolytic activity in nodal mWAT without any contribution of eWAT. The adipocyte‐derived cytokines are not supposed to be involved in enhanced lipolysis. We first demonstrated enhanced presence of epinephrine in perinodal adipocytes, which may contribute to the regulation of local lipolytic activity by auto/paracrine fashion and thus provide independent fuel supply to activated lymph nodes.

Supported by: grant P34, Czech Republic and grant APVV‐15‐0229, Slovak Republic.

**4-05**

**Proteome quantification of *in vivo* and *in vitro* cancer cachexia models**

H. Alexander Ebhardt ^1,6^, Simone Degen^3^, Valentina Tadini^3^, Alain Schilb^3^, Neil Johns^5^, Carolyn A. Greig^4^, Kenneth C. H. Fearon^5^, Ruedi Aebersold^1,2^ and Carsten Jacobi^3^

^1^ *Institute of Molecular Systems Biology, Department of Biology, ETH Zürich, Zürich, Switzerland;* ^2^ *Faculty of Science, University of Zürich, Zürich, Switzerland;* ^3^ *Novartis Institutes for BioMedical Research Basel, Novartis Pharma AG, Basel, Switzerland;* ^4^ *School of Sport, Exercise and Rehabilitation Sciences and MRC‐Arthritis Research UK Centre for Musculoskeletal Ageing Research, University of Birmingham, Birmingham, United Kingdom;* ^5^ *Clinical Sciences (Surgery), University of Edinburgh, Edinburgh, UK;* ^6^ *current address: Systems Biology Ireland, University College Dublin, Dublin, Ireland*

**Background and Aims:** As cancer frequently occurs in old age, identifying and differentiating molecular mechanisms mediating muscle wasting in cancer cachexia vs. age‐related sarcopenia is challenging. We aimed to characterize the underlying mechanism leading to proteome changes in cachectic skeletal muscle.

**Methods:** We used a range of omics approaches: undepleted proteome was quantified using advanced high mass accuracy mass spectrometers in SWATH acquisition mode; phospho epitopes were quantified using protein arrays; morphology was assessed using fluorescent microscopy.

**Results:** We compared the proteome of muscle from healthy elderly people with and without age‐related sarcopenia, with that of muscle obtained from diagnosed cancer and cancer cachexia patients. Using multiple proteomic quantification approaches, we identified a protein signature that is able to identify cancer cachexia patients and differentiate them from the other comparison groups. To relate observed proteome changes in cancer cachexia patients back to underlying molecular mechanisms, we mimicked environmental challenges of muscle regeneration using an *in vitro* system of myogenesis. We investigated changes in the proteome and signal transduction in the presence or absence of tumour necrosis factor‐α as a function of time. We identified aberrations in key proteins of energy metabolism and signaling pathways upon exposure to tumour necrosis factor‐α, which reflect observed changes in human muscle biopsies. Additionally, we compared proteome and morphology dynamics of myogenesis as a function of myoblasts originating from an 83‐year‐old vs. a fetal donor to elicit age‐dependent effects of myogenesis.

**Conclusion:** The work presented here lays the foundation for further understanding of muscle wasting diseases and holds the promise of overcoming ambiguous weight loss as a measure for defining cachexia to be replaced by a precise protein signature.

**4-06**

**Ageing affects regeneration and cell cycle regulation in human skeletal muscle undergoing atrophy and regrowth**

Ulrik Frandsen^1^, Tatyana Prokhorova^1^, Line Jensen^1^, Lars G. Hvid^1^, Peter Schjerling^2^, Per Aagaard^1^, Michael Kjaer^2^ and Charlotte Suetta ^3^

^1^ *Institute of Sports Science and Clinical Biomechanics, SDU Muscle Research Cluster (SMRC), University of Southern Denmark, Denmark;* ^2^ *Institute of Sports Medicine and Center of Healthy Aging, Faculty of Health, University of Copenhagen, Bispebjerg Hospital, Denmark;* ^3^ *Department of Clinical Physiology, Nuclear Medicine & PET, Rigshospitalet, University of Copenhagen, Denmark*

Cellular senescence is an irreversible arrest of cell division, which could influence the regenerative potential of skeletal muscle stem cells (satellite cells) during aging. The molecular mechanism of senescence is complex and involves epigenetic control of the polycomb repressive complexes (PRC1 and PRC2), as well as CDNK2A (p16) and TP53 tumour‐mediated repression of cyclin‐dependent kinases and G1 cell cycle arrest.

**Purpose:** The study sought to investigate the effect of ageing on satellite cell cycle regulation in human skeletal muscle undergoing atrophy and regrowth induced by short‐term immobility and subsequent reloading.

**Methods:** Myofibre atrophy was induced by application of a knee brace for a period of 4 days in young (Y, ~20 years old, *n* = 7–9) and older (O, ~70 years old, *n* = 7–9) individuals. Muscle regrowth after atrophy was induced by 3 days of re‐ambulation supplemented by one session of supervised unilateral resistance training for the disused leg 3 days after brace removal. Muscle biopsies (VL) were collected pre and at 1, 2, and 4 days of immobility and after additional 6 days of re‐mobilization (10 day). Protein and mRNA expression levels of CDNK2A (p16), CDKN1A (p21), CDKN1B (p27), TP53, and PCNA were determined using real‐time reverse transcription polymerase chain reaction and western blotting, respectively. Satellite cell (SC) content was determined by immunohistochemical analysis of Pax7 expression.

**Results:** CDNK2A (p16) mRNA was upregulated at 2d and 4d in O compared to Y and at 10d in Y and O compared to pre (*P* < 0.05). TP53 mRNA was upregulated at 2d in O and at 4d in Y and O compared to pre, while downregulated at 10d in Y and O compared to 4d (*P* < 0.05). CDKN1B (p27) mRNA was downregulated in Y and O at 4d and 10d compared to pre (*P* < 0.05). CDNK2A (p16) protein in Y was lower at 1d‐4d and similar to d10 whereas O was higher at 1d‐4d and d10 compared to pre (*P* < 0.05). PCNA protein was upregulated in Y (5.5‐fold) but blunted in O (1.6‐fold) at 10d compared to pre (*P* < 0.05). Percentage pax7 SC of total myonucli was at pre (Y: 4.8%, O: 4.0 %) and increased in Y (8.5%) and remained unchanged in O (4.8%), *P* < 0.05.

**Conclusion:** CDNK2A (p16) and TP53 early (2–4 days) were selectively up‐regulated during immobility in O compared with Y subjects, suggesting that cellular senescense and SC cycle arrest could be implicated in the defective regenerative response in O compared with Y. Further analysis of epigenetic modifications may provide further explanation for the present findings.

**6-10**

**Nutrition and sarcopenia in frail elderly: a randomized controlled trial of the effects of marine protein hydrolysates to improve physical performance**

Linda Kornstad Nygård ^1^, Ingunn Mundal^1^, Lisbeth Dahl^2^ and Anne Marie Mork Rokstad^1,3^

^1^ *Molde University College, Molde, Norway;* ^2^ *National Institute of Nutrition and Seafood Research (NIFES), Bergen, Norway;* ^3^ *Norwegian National Advisory Unit on Ageing and Health, Norway*

**Background**: As the average life expectancy have increased, age‐related loss of muscle mass (sarcopenia) lead to decline in physical performance, loss of independence and lower quality of life in a large group of the population. Hence, it is important to study how we can delay or prevent progression of sarcopenia. The aim of the study is to assess the effect of a marine protein hydrolysate on sarcopenia‐related outcomes like handgrip strength, estimated leg muscle volume, or gait speed.

**Methods**: This study is a randomized, double‐blinded trial aiming to include 100 eligible patients (≥65 years old) receiving municipal home care service. The intervention group (*n* = 50) will receive three tablets of marine protein hydrolysate two times per day (total 3 g) in 12 months. The control group (*n* = 50) will receive similar looking placebo tablets. Assessments of physical function (SPPB), handgrip strength, estimated leg muscle volume (anthropometric) nutritional status (MNA), dietary intake (24 h multiple‐pass‐recall and a short Food Frequency Questionnaire), and health‐related quality of life (EQ‐5D) will be made in the patients' own home at baseline and after 6 and 12 months of intervention.

**Discussion**: Previous research on health effect of marine proteins is sparse, especially as compared with the comprehensive documentation of the effects of fish oils. The project will also provide useful information on protein intake, nutritional status, and food habits in home‐dwelling elderly. Malnutrition among home‐dwelling elderly may be common, and we need further documentation about its prevalence and causes together with data on dietary intake in this population. This study may provide foundation for future use of bioactive foods, aiming to maintain muscle health. A nutritional supplement that delays or prevents age‐related muscle loss will have potential implications for future need of health care in an ageing population.

Trial Registration number: Clinical Trial Registration‐URL: www.clinicaltrials.gov. Unique identifier: NCT02890290

**6-11**

**Preclinical studies on innovative clinical nutrition**

Gerben C. M. Zondag^1^, Anita M. van den Hoek^2^, Robert Kleemann^2^, Annemarie Rietman ^1^ and Rein Strijker^1^

^1^ *Vitalnext BV, Leiden, The Netherlands;* ^2^ *Department of Metabolic Health Research, Netherlands Organization for Applied Scientific Research (TNO), Leiden, The Netherlands*

**Background**: Vital01 is a novel, proprietary clinical nutrition comprising an optimized protein composition, high concentration of free branched chain amino acids, vitamin D, and ursolic acid. It aims to drastically improve the treatment of malnourished patients.

**Methods:** To determine whether Vital01 has benefits over existing oral nutritional supplements (ONS), we conducted a study in malnourished mice. Healthy adult mice were calory restricted until they lost 25% of body weight. Next, these mice were fed regular animal feed (RAF) or animal feed supplemented with Standard ONS or Vital01. Treatments were normalized on either caloric or protein content.

**Results:**

- Refeeding with Vital01 resulted in a statistically significant faster weight gain compared with standard ONS or RAF. In addition, lean body mass (indicative for muscle mass) also increased faster in Vital01‐fed animals than in RAF or standard ONS‐fed animals (supported by analysis of isolated muscle tissue).
- Transcriptome analysis of muscle tissue showed that Vital01, in comparison with RAF and ONS, enhanced the IGF pathway (involved in muscle growth) and reduced Atrogin1 expression (involved in muscle degradation).
- Inhibitory effects on muscle degradation could be attributed to the presence of ursolic acid.

Notably, ursolic acid also activated pathways involved in immune surveillance and inflammatory responses, potentially leading to a protective health effect.

**Conclusion:** Vital01 is a new clinical nutrition that rapidly restores body weight and improves gain of muscle mass in a more efficient manner than current treatments. It also activates pathways that are indicative for a proper functioning of the immune system.

**Next steps:** In 2016, a comparable clinical trial in human malnourished patients will be initiated in collaboration with Wageningen University (NL). Trial design and initial results will be incorporated in our presentation.

**6-12**

**Study protocol. Validation of malnutrition parameters and indication for nutritional treatment in nursing home. The NuTrIR Study (Nutritional Treatment Indication in Residents Study)**

Vincenzo Malafarina ^1,2^, Fernando Gomez‐Busto^3^, Sonia Cabrerizo^4^, Virginia Andia Muñoz^4^, Naiara Fernández‐Gutierrez^5^ and Iñaki Artaza‐Artabe^5^

^1^ *Department of Nutrition, Food Science and Physiology, School of Pharmacy, University of Navarra, Pamplona, Spain;* ^2^ *Department of Geriatrics, Complejo Hospitalario de Navarra, Pamplona, Spain;* ^3^ *Department of Geriatrics, Residencia San Prudencio, Ayuntamiento de Vitoria‐Gasteiz, Alava, Spain;* ^4^ *Residencia San Prudencio, Ayuntamiento de Vitoria‐Gasteiz, Alava, Spain;* ^5^ *Department of Geriatrics, Orue Centro Socio Sanitario, Grupo Igurco, Amorebieta, Bizkaya*

**Background and Aims:** The prevalence of malnutrition increases with age, because of factors such as comorbidity, loss of appetite, reduced physical activity, poor oral health, and inability to eat independently, or require assistance for this and cognitive impairment . The prevalence of malnutrition in nursing home is between 6.5% and 85%.

The aim is to validate malnutrition criteria specific to nursing home.

**Hypothesis:** The hypothesis is that the criteria currently used for indication of oral nutritional supplementation pose a risk of infra‐treat malnourished elderly because of the limitations in identifying the correct indication of nutritional therapy.

We therefore expect to find in malnourished elderly who do not receive nutritional treatment, a worsening of both malnutrition and functional status.

**Methods:** A prospective study with 4 month follow‐up was conducted. Subjects living in nursing homes will be included, ≥65 years with MNA‐SF <11. Residents receiving tube feeding, those with stage 7 of the global deterioration scale of Reisberg, and those in palliative treatment will be excluded. Both the weight (kg) and height (cm) were collected, and body mass index was calculated. Both the type of diet and total calories are also collected. The basal intake will be recorded for 72 h, and it will be repeated at the end of the study. A blood sample is collected for haemoglobin and white blood cells, total proteins, albumin, C‐reactive protein, total cholesterol, triglycerides, and 25(OH)D.

For autonomy in activities of daily living, Barthel index (zero, totally dependent, and one‐hundred, completely independent) will be used. Charlson index will be used to calculate comorbidity.

For cognitive study, the Mini Mental State Examination will be used.

The study protocol has been prepared in accordance with the requirements of Good Clinical Practice of the European Union and the current revision of the Declaration of Helsinki and will be submitted to the Ethics Committee on Social Research of the Provincial Council of Bizkaia.

**Conclusion:** Recruitment has started in January 2016, and it will end in October 2016.

**6-13**

**Anorexia, functional capacity, and clinical outcome in patients with chronic heart failure: results from the Studies Investigating Comorbidities Aggravating Heart Failure (SICA‐HF)**

Masakazu Saitoh ^1^, Amir Emami^1^, Junichi Ishida^1^, Nicole Ebner^1^, Miroslava Valentova^1^, Anja Sandek^1^, Wolfram Doehner^2^, Stefan D. Anker^1^ and Stephan von Haehling^1^

^1^ *Innovative Clinical Trials, Department of Cardiology and Pneumology, University Medical Centre Göttingen, Göttingen, Germany;* ^2^ *Centre for Stroke Research Berlin, and Department of Cardiology, Virchow Klinikum, Charité‐Universitätsmedizin Berlin, Berlin, Germany*

**Objectives:** Anorexia has implications for the development of malnutrition, and it has been associated with poor outcomes in elderly people or in patients with chronic disease. Moreover, anorexia is one of the causes of impaired functional capacity. Our objective was to assess the determinants of anorexia and to evaluate the associations of anorexia with functional capacity and outcomes in ambulatory patients with heart failure (HF).

**Methods and Results:** We assessed the presence of anorexia in 166 patients (25 female, 66 ± 12 years of age) who were recruited at the Charite Medical School, Campus Virchow‐Klinikum, Berlin, Germany, as part of the Studies Investigating Co‐morbidities Aggravating Heart Failure (SICA‐HF). Anorexia was assessed by a 6‐point Likert scale (ranging from 0 to 5) and defined as patients with presence of anorexia. Cachexia was defined as the presence of non‐oedematous, non‐intentional weight loss of ≥5% over a period of at least 6 months. Functional capacity was assessed as peak oxygen consumption (peak VO_2_) by cardiopulmonary exercise testing, 6 min walk testing, and short physical performance battery test. A total of 57 patients (34%) presented with anorexia, and these patients with anorexia indicated lower values of peak VO_2_, 6 min walk distance, and short physical performance battery score (all, *P* < 0.05). Using multivariate analysis, high‐sensitivity C‐reactive protein [odds ratio (OR) 1.240, 95% CI 1.007–1.527, *P* = 0.043], diuretics use (OR 5.759, CI 1.207–27.466, *P* = 0.028), and cachexia (OR 2.532, CI 1.036–6.198, *P* = 0.042) turned out to be an independent predictor of anorexia in patients with HF. A total of 22 (13%) patients died during a mean follow‐up of 22.5 months. Kaplan–Meier curves for cumulative survival showed that those patients with anorexia presented higher mortality rates within 24 months (log‐rank test, *P* = 0.029).

**Conclusions:** Inflammation, diuretics use, and cachexia were associated with anorexia in patients with HF, and these patents with anorexia showed impaired functional capacity and outcomes. The evaluation of nutritional habit should be included as a fundamental part in the overall assessment of patients with HF.

